# Living with chronic pain and returning to work: lessons learnt from a process evaluation embedded in the ReISE trial

**DOI:** 10.1136/bmjopen-2026-125090

**Published:** 2026-10-22

**Authors:** Pernille Marie Stahr Irgens, David R Ellard, Martin Underwood, Margreth Grotle, Jakob Thor Einar Holmgard, Pål Andre Amundsen, Magnus Højen, Kim Burton, Robert Froud

**Affiliations:** 1 Oslo Metropolitan University Faculty of Health Sciences, Oslo, Norway; 2 Faculty of Health Sciences and Technology, Kristiania University of Applied Sciences, Oslo, Norway; 3 Clinical Trials Unit, University of Warwick, Coventry, UK; 4 University Hospitals Coventry and Warwickshire NHS Trust, Coventry, UK; 5 Department of Physiotherapy, Oslo Metropolitan University, Oslo, Norway; 6 Research and Communication Unit for Musculoskeletal Health, Oslo universitetssykehus Ulleval, Oslo, Norway; 7 Norwegian Back Pain Association, Drammen, Norway; 8 Work & Health Consultant, Liverpool, UK

**Keywords:** Workplace, PAIN MANAGEMENT, REHABILITATION MEDICINE

## Abstract

**Objectives:**

To evaluate implementation of a supported work-placement intervention for unemployed people with persistent pain, examining reach, dose, fidelity, acceptability, candidate mechanisms and contextual factors to inform interpretation of the trial results.

**Design:**

Mixed-methods process evaluation conducted alongside a pragmatic, cohort-randomised controlled trial, following UK Medical Research Council guidance and Linnan and Steckler’s components.

**Setting:**

Kristiania University of Applied Sciences in Norway.

**Participants:**

Adults aged 18–65, unemployed for at least 1 month, with pain for more than 3 months and wanting to return to work. The study cohort included 447 participants, and the participants included in this process evaluation were those randomly sampled to be offered the intervention. Quantitative process data covered all invitees. Qualitative data comprised interviews with 30 purposively sampled participants, including those who declined, and the intervention staff (five case managers, the work familiarisation facilitator, the placement recruiter and the trial coordinator).

**Interventions:**

A supported work-placement intervention with individually tailored biopsychosocial case management, a 2-day work familiarisation session and a 6-week supported work placement.

**Outcome measures:**

Process indicators of reach and recruitment, dose delivered and received, fidelity, acceptability, candidate mechanisms of impact and contextual factors drawn from trial and case-manager records, questionnaires, fidelity observation and interviews.

**Results:**

Of 223 participants offered the intervention, 83 (37%) consented and 119 (53%) declined. Among those consenting, 93% completed the initial assessment, 73% the work familiarisation session and 27% the 6-week placement. Attrition ranged from 40% to 51% across the case managers. 3 of 14 work familiarisation sessions involving 12 participants were observed, with a mean adherence of 99% and competence of 100%. The case-manager relationship emerged as the central candidate mechanism linking intervention exposure to engagement and perceived readiness to work, but progression depended on alignment between participant readiness, welfare-system conditions and labour-market opportunity.

**Conclusions:**

Core components were delivered with high documented fidelity, and the intervention was acceptable to those who engaged. Uptake and progression were constrained by participant heterogeneity, system conditions and limited integration between components. Future delivery should use assessment of support needs to guide timing and tailoring, alongside timely placement and closer welfare-system coordination.

**Trial registration number:**

ISRCTN registry: 85437524 Registered 30/03/2022. The ReISE protocol is registered with the Norwegian Research Council (grant number 326732/ABHO).

**STRENGTHS AND LIMITATIONS OF THIS STUDY**

- A prespecified, protocol-driven process evaluation, grounded in Medical Research Council guidance and an explicit logic model, supported methodological rigour and transparency.
- Multiple data sources and informants enabled triangulation across participant, case manager, facilitator, placement recruiter and trial coordinator perspectives.
- Prospective process data and direct observation of work familiarisation sessions strengthened the assessment of delivery, although case-management fidelity relied largely on documented forms rather than observation.
- Including participants who declined the intervention clarified reach and acceptability, although the interview sample was predominantly female and drawn mainly from those who engaged.
- Conducting the evaluation in a single trial setting limited transferability to systems with different welfare structures or labour markets.

## Introduction

 Persistent pain, most often of musculoskeletal origin, is the leading contributor to years lived with disability worldwide.^1 2^ In Norway, it accounts for the largest share of long-term sickness absence and substantial share of lost workdays and socioeconomic costs.^3–5^ Because work supports health and financial security while worklessness harms both, sustained return-to-work matters for this population.^6–9^

Return-to-work, however, is not a discrete event. Qualitative syntheses across chronic conditions show it to be multifactorial and multistakeholder,^10 11^ requiring a holistic, person-centred approach with a designated coordinating role across healthcare, workplace and welfare services, rather than symptom management alone.^10 12^ In ReISE, the case manager was intended to provide this coordinating function. This is harder still for people who are unemployed as they have no fixed workplace and depend on long-term public income-replacement benefits administered by the Norwegian Labour and Welfare Administration. Multidomain, case-managed interventions combining a health focus, service coordination and work accommodation can support return-to-work.^13 14^ Supported approaches, such as Individual Placement and Support (IPS), have shown limited, inconsistent effects outside mental health populations.^15 16^

Attempting to address this unmet need, the ‘Returning people with persistent pain to work using Individual Supported Employment placement’ (ReISE) study adapted and tested in Norway, in a cohort randomised approach, the effectiveness of an intervention that showed promising results in the UK.^17^ The intervention comprised biopsychosocial case management, in which case managers used the Psychosocial Flags Framework to identify obstacles to work and develop a return-to-work plan, a work familiarisation session and a supported work placement.^18 19^ An internal pilot supported progression to the full trial, while identifying low intervention uptake and difficulties securing timely work placements.^17 20^ Progression was endorsed with a recommendation to increase the number randomised to intervention and to identify additional placement sources.

While trials can establish if interventions are effective, they provide limited information about how delivery, mechanisms and context shape outcomes.^21^ Embedded process evaluations address this gap and crucially distinguish failure of an intervention’s concept from failure of its execution.^21–26^

The aims of this process evaluation were to assess recruitment, reach, dose, fidelity and acceptability of the ReISE intervention; capture participant and staff experiences and perspectives and explore the candidate mechanisms, pathways and contextual factors in relation to the underlying assumptions of the process evaluation.

## Methods

### Study design and process evaluation framework

This mixed-methods process evaluation was conducted alongside the ReISE trial, combining quantitative and qualitative data in line with UK Medical Research Council (MRC) guidance for process evaluations of complex interventions.^21^ The report followed the Consolidated Criteria for Reporting Qualitative Research (COREQ) (online supplemental table S1, online supplemental file 1). Participants first entered a longitudinal observational cohort, where a random subsample was offered the intervention. The allocation ratio was changed during the trial (initially 1:3, then 1:2 during the pilot study, and again to 2:1 during the study) in response to lower-than-anticipated acceptance of intervention invitations. The trial design and randomisation are reported in detail in the study protocol and online supplemental file 1, part 1.^18^ All participants invited to the intervention, whether they accepted or declined, were eligible for the process evaluation.

The evaluation was structured around the process evaluation components proposed by Linnan and Steckler: reach, dose and fidelity, together with acceptability, candidate mechanisms of impact and contextual factors from the UK MRC guidelines.^21 27^ The schedule of enrolment, intervention delivery, process data collection and follow-up assessments is shown in figure 1. User representatives from the Norwegian Back Pain Association were involved in and consulted on developing the research question and outcome measures, and recruitment strategy.

**Figure 1:**
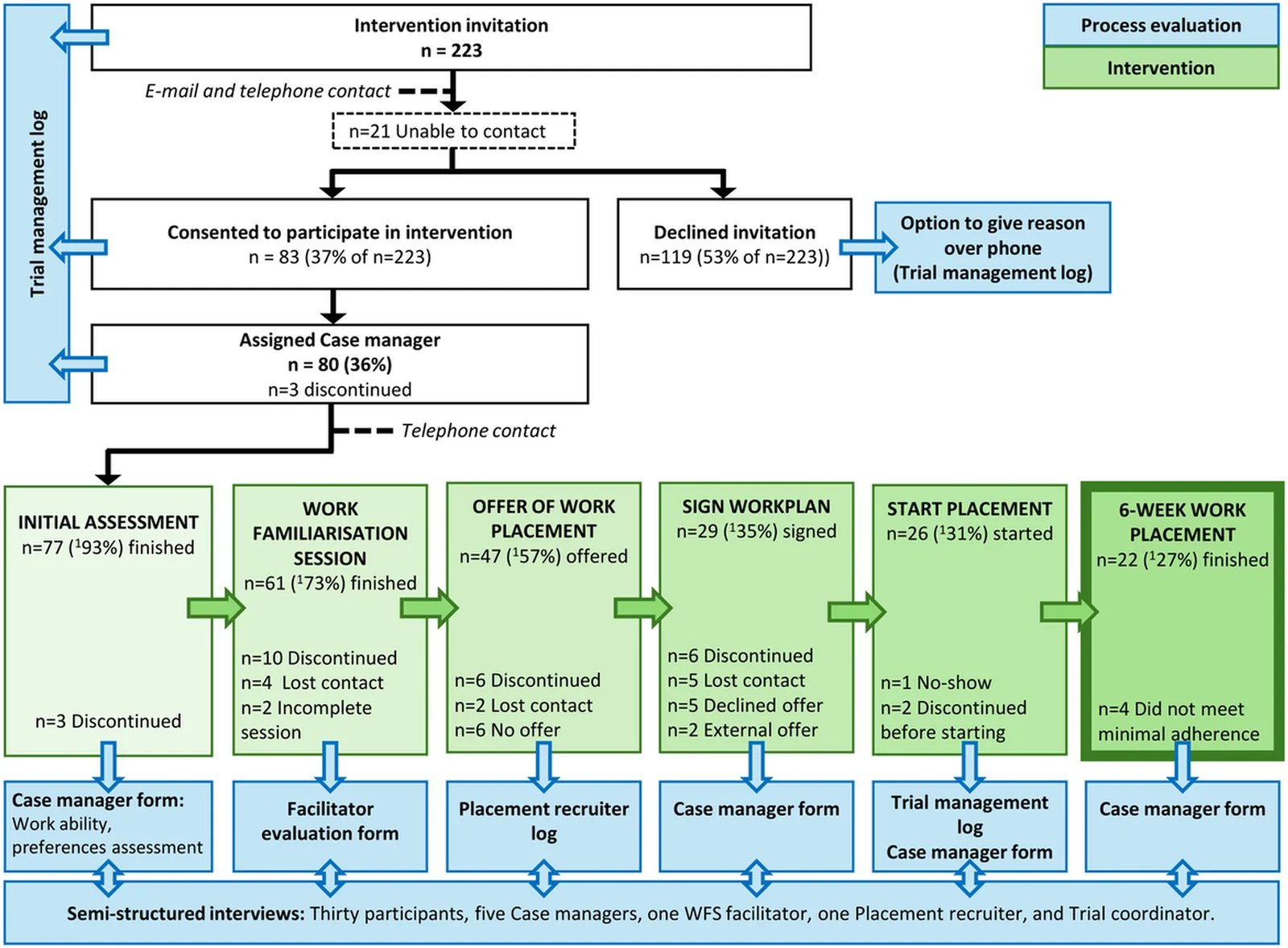
Flow of participants after randomisation to intervention to completed work placement. Green boxes show the intervention flow and reasons for attrition. Blue boxes show the data sources for the process evaluation content. ^1^Of n=83 consenting to participate in intervention. WFS, work familiarisation session.

### Setting, intervention and participants

The study recruited participants nationally in Norway and comprised individually tailored case management, a 2-day work familiarisation session and a 6-week supported work placement. Full details are provided in the trial protocol and online supplemental file 1, part 1.^18^ In brief, participants who accepted the intervention invitation were allocated to a trained case manager. Case managers were clinicians experienced in persistent pain and received training in the Psychosocial Flags Framework (for detailed description, see online supplemental file 1, part 1.2), work-focused pain management, how to handle adverse events, the Norwegian benefits system and project-specific tools.^19^ The case manager conducted an initial work-focused assessment and supported development of a return-to-work plan. Participants were then invited to a work familiarisation session, delivered in small groups where possible, to prepare for work re-entry. A placement recruiter identified potential work placements, representing an adaptation from the original protocol after the original collaborating recruitment agency proved unworkable. When a placement was identified, the participant, case manager and placement recruiter agreed a workability plan specifying tasks, hours, support needs and accommodations. Case managers provided follow-up during the intervention, primarily by telephone, video or text message.

Trial eligibility and randomisation are described in the protocol.^18^ Eligible participants were aged 18–65, unemployed for at least 1 month, had experienced pain for more than 3 months and wanted to return to work. Consent to the trial was obtained either digitally or in writing, with identity confirmed through BankID verification (a Norwegian national electronic identification system) and a paper consent option available. A voucher of NOK 200 (2022 equivalent GBP 16.90, USD 20.90, EUR 19.80) was offered for joining the cohort, with no additional payment for accepting the intervention invitation. Travel expenses for work familiarisation sessions and work placements were eligible for reimbursement. The qualitative component drew on two informant groups that were analysed separately throughout: trial participants and intervention staff. 30 participants invited to the intervention were purposively sampled after their 12-month follow-up, structured by gender, age, whether they accepted or declined the invitation and the intervention stage reached. Interviewee characteristics are presented at group level to reduce the risk of identification through combinations of characteristics and quotations. All eight intervention-staff were invited to interview: the five case managers, the work familiarisation session facilitator, the placement recruiter and the trial coordinator.

### Data sources and collection

The quantitative process evaluation included all participants formally invited to the intervention group. Recruitment and reach were described using expression-of-interest, screening, consent, randomisation and intervention-invitation records, together with participant-reported recruitment source. Participant characteristics were drawn from questionnaires completed at baseline and at 3, 6 and 12 months, administered through Nettskjema (University of Oslo, Norway) or on paper and stored in an electronic case report form, and included quality-of-life and work-related measures such as work ability and confidence in returning to work. Case managers completed a structured form (Case Manager Form) for each participant recording the core intervention components, including frequency, duration, dose and content of support sessions. Attendance, placement and trial management records stored in an electronic trial management form provided further quantitative process data.

Qualitative data contextualised the quantitative findings.^28^ The principal source of data was semi-structured, one-to-one interviews. Additional qualitative data comprised Case Manager Form, which included correspondence between case managers and both participants and other staff. Participants could be interviewed by telephone or video, with verbal consent recorded at interview start. No incentive was offered for participation in the process-evaluation interviews. Interviews explored prior experiences of the health and welfare system, expectations of and experiences during the intervention, including acceptability and perceived usefulness, and personal and organisational contextual factors (topic guide summaries, (online supplemental tables S2 and S3 and online supplemental file, part 2). Interviews were audio-recorded using Nettskjema Diktafon (University of Oslo, Norway), stored in Services for Sensitive Data (TSD, University of Oslo), transcribed verbatim using Whisper (OpenAI, San Francisco, California, USA), checked manually for accuracy and de-identified. Staff were interviewed individually about experiences of delivering the intervention, factors affecting delivery and suggestions for improving implementation.

### Process evaluation components

Figure 2 shows how the process evaluation domains relate to the intervention logic, including data sources. Informed by the Standards for Reporting Implementation studies and the MRC guidelines and adapted from Nichols *et al*, Trials, 2019, which is distributed under the creative commons licence^21 29 30^ (http://creativecommons.org/licences/by/4.0/).

**Figure 2:**
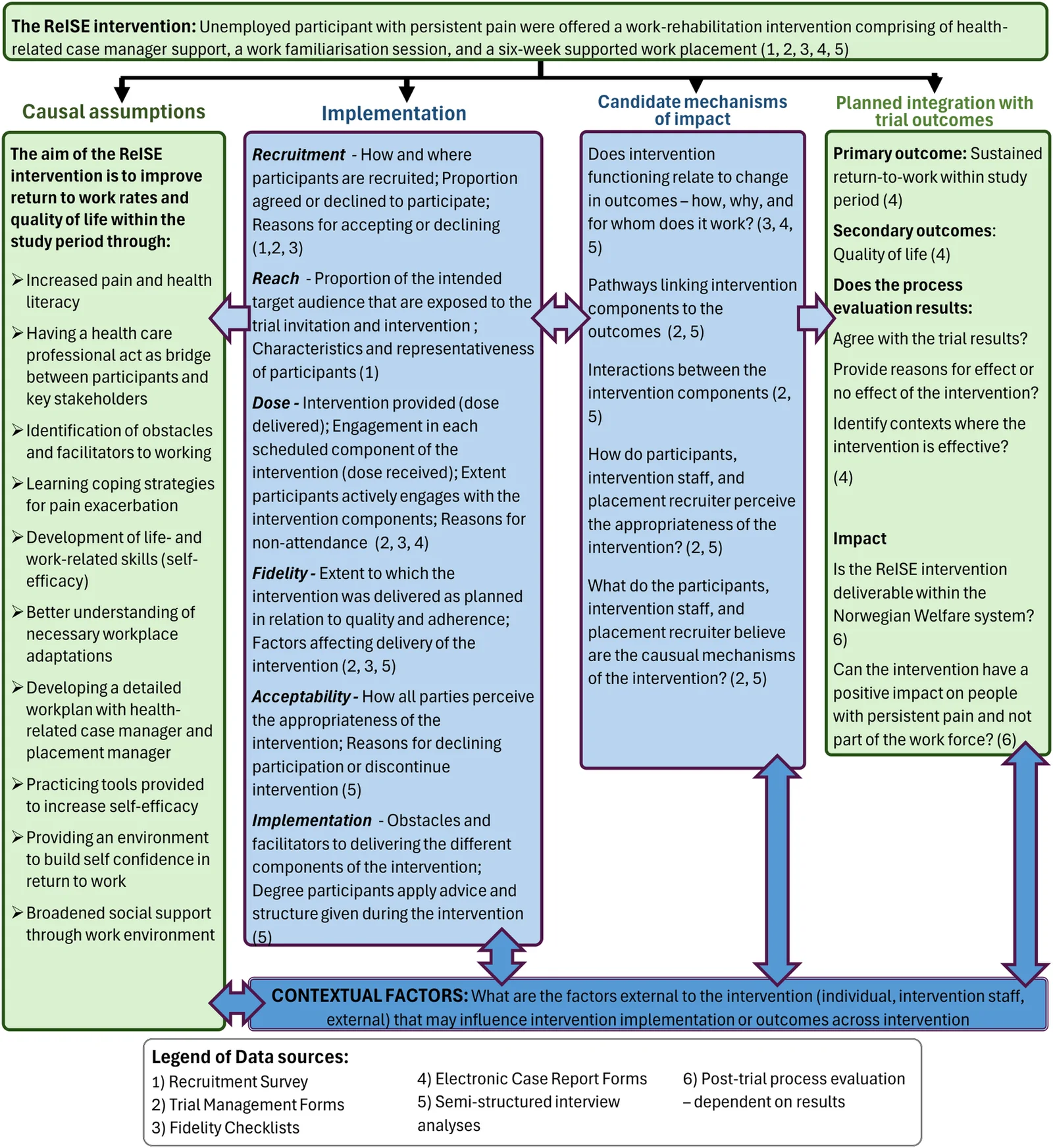
Boxes in blue represent the process evaluation content. Questions requiring trial outcome data, including those shown under ‘Planned integration with trial outcomes’, represent planned subsequent work and are not addressed in this report. Informed by the Standards for Reporting Implementation studies and the MRC guidelines and adapted from Nichols *et al*, Trials, 2019, which is distributed under the creative commons licence.^21 29 30^ (http://creativecommons.org/licences/by/4.0/). MRC, Medical Research Council.

The sections below describe how each domain was operationalised and analysed.

Reach and recruitment were assessed by describing who was reached, numbers of eligible and included participants, recruitment routes (study advertisement, social media or health and work-related services) and how the recruited sample related to the intended population. Recruitment strategies and barriers were examined in relation to logistical and eligibility challenges, participant preferences and intervention invitation outcomes.

Dose was assessed as dose delivered (the amount of intervention provided) and dose received (the amount taken up by participants) using Case Manager Form, Trial management form attendance records, placement recruiter logs and participant interview accounts. Timing between intervention steps described progression through the pathway and compared with the trial protocol.^18^ Attrition, defined as dropping out or being lost to follow-up, was calculated from Trial management forms and the Case Manager Form.

Fidelity was defined as the extent to which the intervention was delivered as planned.^21 31^ For case management, a fidelity checklist extracted from the Case Manager Forms and based on the training manual (for details, see online supplemental file 1, part 1.2), recorded adherence to forms, whether a return-to-work plan was made, and factors affecting delivery; case manager-participant correspondence was included in qualitative analysis. For the work familiarisation session, an observer assessed a purposive sample of three sessions using a checklist of facilitator adherence to planned content and competence in delivery (calculated percentage score; online supplemental table S5, online supplemental file 1, part 4). For the work placement, fidelity drew on workability plans, placement recruiter logs, and records of adaptations to placement recruitment. Fidelity was described through synthesis of qualitative and quantitative data.

Acceptability was assessed through participant and staff accounts of the appropriateness and perceived usefulness of the intervention and its components, drawing on interviews and documented telephone contacts in the Trial management forms. Candidate mechanisms of impact were explored primarily through qualitative data, examining how all intervention participants and stakeholders described pathways linking intervention components to engagement, perceived change and work-related or health-related outcomes; these accounts were treated as candidate rather than established mechanisms given the evidential limits of the available data. Contextual factors included staff capacity, intervention coordination, participant readiness, health and welfare-system experiences, benefit-related concerns, employer engagement, and labour-market constraints.

### Data analysis and synthesis

Quantitative process data were summarised descriptively. Normally distributed continuous data were reported as means with SD, non-parametric data as medians with IQRs, and categorical data as counts and percentages.

Qualitative interview and observational data were managed in NVivo (QSR International, Australia) within the TSD environment and analysed using the Framework method, combining deductive coding based on the process evaluation domains with inductive coding from emerging themes, suited to an evaluation with predefined domains requiring comparison across pathways, stages, and stakeholder roles.^32^ A subset of transcripts was independently double-coded, with differences resolved with a third author by consensus; data were then charted into framework matrices.^33^ At the interpretation stage, following completion of the separate quantitative and qualitative analyses, qualitative themes were compared with quantitative process indicators drawn from the trial and case manager records to identify convergence and divergence. The process evaluation domains provided a common structure for the quantitative indicators and qualitative framework, allowing comparison domain by domain. For example, the recorded intervals between the work familiarisation session and a placement offer were considered alongside participants’ accounts of delay and staff accounts of the difficulty of finding suitable placements. Staff and participant data were treated as complementary accounts of different positions in the intervention pathway rather than as interchangeable sources, and where the two diverged both readings were retained rather than reconciled. Convergence and divergence were judged by discussion between PMSI and DRE. The comparisons were used to assess whether implementation challenges reflected intervention design, delivery conditions, participant readiness or contextual constraints outside the control of the delivery team, thus preserving the distinction between implementation failure, limited intervention exposure and contextual constraint. Analysis, drafting and submission of this paper were completed before trial outcome results were available.

## Results

### Recruitment to the cohort and intervention group

Cohort recruitment took place between June 2022 and June 2024. Figure 3 illustrates the flow of participants from the 1622 individuals accessing the expression of interest form through to the 223 participants randomised to the intervention group. Recruitment was open nationally and relied mainly on self-referral, rather than selection from an enumerated list of eligible people. The 1622 individuals therefore represent people accessing the expression of interest form. Available national statistics do not identify the number meeting all ReISE eligibility criteria, and the proportion of the eligible population reached could not be calculated.

**Figure 3:**
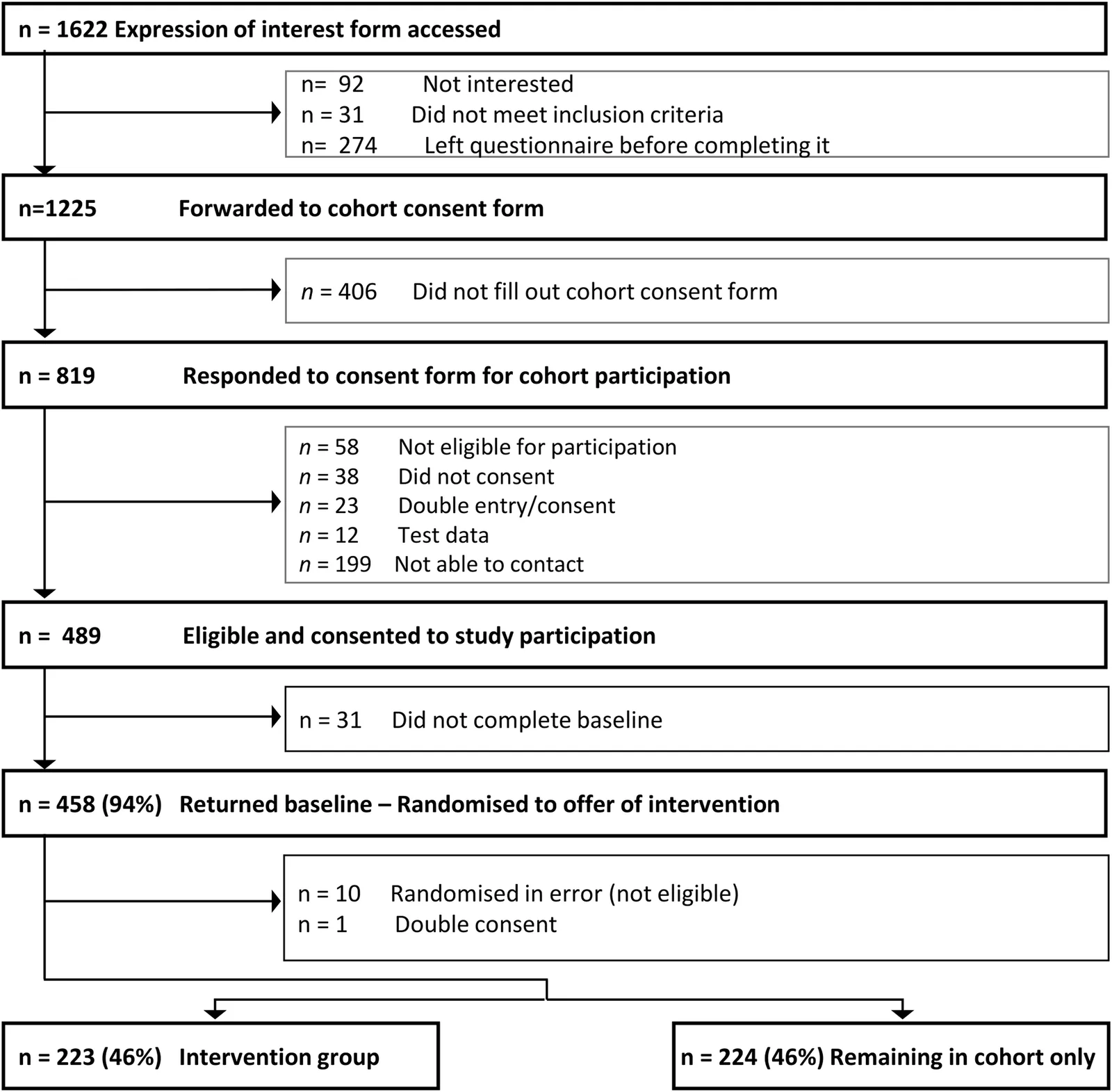
Recruitment flow from access to the expression of interest form through screening, consent, baseline completion and allocation to an intervention offer or cohort-only follow-up.

Among the 223 formally invited to the intervention group, 83 (37%) consented to participate, 119 (53%) declined and the remainder either could not be contacted, withdrew or were lost to follow-up (figure 1). Invitees who accepted and those who declined or could not be contacted were similar in age, sex and duration out of work (online supplemental table S4, online supplemental file 1, part 3). Those who declined or could not be contacted reported lower work ability on a 0–10 point scale (2.3 vs 3.1) and confidence in return to work (3.6 vs 4.8), and more often expected return to work to take longer than 10 months (82% vs 71%). Pain lasting more than 10 years was more common among those who accepted (65% vs 51%).

The reach and recruitment process was resource-intensive: the majority required three or more contact attempts before responding, with the final call lasting 15–30 min. Table 1 shows the time intervals between each intervention stage. The increase in time from invitation to participant decision partly reflected the requirement for additional consent procedures, including BankID verification.

**Table 1:** Time intervals between each of the intervention steps

Time interval (days)	n	Median (IQR)	Min	Max
Baseline to invitation	207	6 (4–14)	0	207
Invitation to response	131	10 (2–43)	0	93
Acceptance to IA	74	18 (11–31)	2	92
IA to WFS	60	19.5 (8–39.5)	−11	130
WFS to offer	46	34 (6–84)	−39	190
Offer to workplan	29	14 (2–35)	0	161
Workplan to start	26	7.5 (6–14)	0	70
Baseline to finish, total time	26	172.5 (133–220)	86	247

Negative values indicate cases where a later process step was completed before the preceding step. This could be due to scheduling conflicts or waiting for a work familiarisation session to fill up with enough participants or for a suitable time to be found. In the situation where this took too long, the participant was given the work familiarisation session on a 1-to-1 basis as a single session lasting about 3 hours.

IA, initial assessment; WFS, work familiarisation session.

Recruitment occurred primarily through social media self-referral, with most participants reporting social media advertisements (41%) or groups (40%) as their source. Recruitment via healthcare services, the Norwegian Labour and Welfare Administration or other formal channels was minimal. The majority of respondents (85%) preferred digital contact for questionnaires.

Full details of recruitment flow, exclusions and procedural challenges are provided in online supplemental file 1, part 1.1 and part 5.

### Interviewees: intervention staff and study participants

The project started with three case managers. A fourth was recruited and trained after approximately 1 year as recruitment volume increased, and a fifth was added during the final 8 months to manage workload and reduce the time between recruitment and intervention start. All the case managers contributed to the interviews, as did the work familiarisation session facilitator, the placement recruiter and the trial coordinator. Only two staff, one case manager and the work familiarisation session facilitator, were interviewed digitally due to geographical distance. The rest were interviewed in person.

30 study participants from those randomised to the intervention gave consent and were interviewed. 11 of these interviewees had declined the intervention invitation. All the interviews were conducted remotely; 11 participants preferred a telephone call to a video call. Interviewed participants were predominantly women and aged 40 years or older. Most reported long-standing pain and prolonged work absence, and baseline work ability and confidence in return-to-work were generally low. All but three expected return-to-work to take more than 10 months. Participants ranged from those rejecting the invitation to those completing the intervention. Further details on interviewee characteristics are provided in online supplemental table S5, online supplemental file 1, part 3.

### Dose

#### Dose delivered: case manager component

Participants were allocated to a case manager within 0–4 days of consenting to the intervention. Scheduling availability was the most common reported cause of delay between acceptance and completed initial assessment (table 1). Seven initial assessments were conducted physically at Kristiania University of Applied Sciences; the remaining 70 were conducted digitally via telephone, SMS or video meeting (table 1).

#### Dose delivered: work familiarisation session

The work familiarisation session comprised a 2-day group session with a planned group size of 5–7 participants. 14 sessions were delivered; the intended group size was achieved in three sessions, five were conducted one-to-one due to scheduling conflicts for the participant or periods of slow recruitment, and the remainder included 2–4 participants. Two sessions were held in person at Kristiania University of Applied Sciences, with the others delivered digitally. In the situation where the duration of finding a placement was very short, a participant was given a single, intensive 1-to-1 session covering the full 2-day course material and exercises, lasting about 3 hours.

#### Dose delivered: work placement offers

One participant was placed through the original recruitment partner before the placement recruiter took over. The median number of employer contacts made per participant was 12 (IQR 8–16). The majority of placements offered were within retail and care/community settings. The time interval between work familiarisation session and receiving a placement offer was the longest of the intervention due to difficulties finding acceptable placements.

#### Dose received (intervention participants)

Figure 1 illustrates the flow of participants through the intervention stages, and the number and causes of participants not completing each stage. Table 2 shows the dose received. 16 participants had only one or two contact points with their case manager as they exited the intervention before work familiarisation session. The majority of the case manager contacts with participants were initiated by the case manager, with 8% initiated by participants. Email contact was minimal at 2%. Of the 63 participating in the work familiarisation session, 61 completed the full 2 days (of which two participants received the intensive, 1-to-1, single session), while two attended only the first of the two scheduled days. While 6 of 47 participants received two placement offers, 6 received no offer despite an active search. All who commenced placement completed a closing evaluation. Of the 26 participants who started a placement, 4 withdrew before 2 weeks and 22 completed the full 6-week placement. No participant discontinued between 2 and 6 weeks; the numbers meeting the minimum 2-week adherence criterion and completing the full placement therefore coincided. Only one participant applied for travel expense reimbursement. Attrition occurred at several points, including failure to sign a workplan, discontinuation, loss to follow-up and declining placement offers.

**Table 2:** Dose received of the participants randomly sampled to be offered the intervention

Dose	Median (IQR)
Number of intervention invitation contact attempts before response (*of n=223) (n)	3 (2–5)
Number of contacts between CM and participant (*of n=80) (n)	10 (1–29)
During placement only (n)	6 (3–15)
Total duration of contact between CM and participant (*of n=80) (hrs)	7 (3–9)
Number of employer contacts made per participant (*of n=61) (n)	12 (8–16)
CM participant attrition rate (*of n=80) (%)	46 (46–51)

* See figure 1 for number of participants in each stage.

CM, case manager.

### Fidelity

Fidelity to the case manager component was high, with all required assessment fields completed and return-to-work plans documented for all participants. Case managers reported that training provided a strong theoretical foundation but was insufficient in practical application. Minor protocol adaptations included direct communication between the placement recruiter and case manager, bypassing the trial coordinator and early placement offers for six participants prior to work familiarisation session completion due to timing constraints.

PMSI observed both days of 3 of the 14 work familiarisation sessions delivered. They were all digital, and included 3, 4 and 5 participants, respectively. Across these observations, mean adherence to the planned content was 99% and competence was 100% (see online supplemental table S5, online supplemental file 1, part 4). These scores describe facilitator delivery in the observed sample only. One planned element addressing pain-related misconceptions regarding work (that pain is often caused by work, means injury or underlying illness, is cured by medical intervention, worsens with work, sick-leave is necessary; and that one should not start work until all pain is gone) was removed after the first two sessions after provoking participant distress and irritation rather than reassurance. Occasional drift from solution-focused discussion towards system-related frustration was noted during observation, but the facilitator was able to bring the discussion back to the planned topic, so this did not affect adherence scores. Due to scheduling issues, one participant attended the work familiarisation session before the Initial Assessment and six participants received a placement offer before attending the work familiarisation session. Minor logistical issues with materials and online delivery were resolved without impacting session flow.

In the placement component, an adaptation was made after the placement agency proved unworkable, which assigned placement search responsibility to a dedicated placement recruiter rather than the original collaborating recruitment agency, which improved efficiency.^18^ The placement recruiter and case manager began communicating directly rather than routing communication through the trial coordinator, with the aim of reducing delays in arranging placements. Employer engagement strategies were adapted to increase participation by emphasising flexibility rather than upfront commitment. All participants commencing placement had completed a workplan. Among those not completing the full placement, reasons included health limitations, lack of meaningful work tasks and unexplained discontinuation.

### Interviews

The interview results below are presented under seven headings comparing the views of the different stakeholders; participants (P), case managers (CM), work familiarisation session facilitator (WFS), placement recruiter (PR) and trial coordinator (TC) involved. These results contribute to the assessment of acceptability, candidate mechanisms of impact and contextual factors, and each heading is discussed in relation to the process evaluation domain it addresses. More detail including more examples can be found in online supplemental file 2, online supplemental table S6.

#### Role of the case manager: central but differently experienced

Both participants and case managers identified the case manager relationship as the most critical element of the intervention. Participants emphasised feeling seen, understood and supported, often contrasting this with prior experiences in the Norwegian Labour and Welfare Administration and healthcare.

He asked good questions… and he understood me. (P10)

But it was really good to sort of be allowed to be frustrated. It was very good… to him, it was okay that I was emotional and fatigued. (P20)

Case managers framed their role as building a working alliance through trust, warmth and time investment, with the scope of engagement frequently surpassing what the intervention design had anticipated.

The aim (was) to achieve some kind of contact and relationship and trust. (CM1)

The job was much bigger… it became a large part of this person’s entire life. (CM4)

There is a strong alignment on the centrality of the relationship as a mechanism of change. However, case managers experienced role ambiguity and scope expansion, while participants primarily evaluated relational quality rather than structural clarity.

#### Need for individualisation versus structural constraints

Participants valued adaptation to individual needs and experienced rigidity as a barrier.

There have been times when you needed five more minutes, and then you were turned away, so to speak. That stopped me a little. (P05)

Case managers also highlighted the importance of flexibility but described constraints imposed by protocols, unclear guidance and availability issues.

You cannot have a script that says this should apply to everyone. (CM2)

Participants attributed lack of adaptation to delivery, whereas case managers linked this to limitations in the intervention design and training. There was agreement, however, that individual tailoring was essential.

#### Participant readiness, motivation and expectations

At cohort entry, participants had confirmed a wish to return to work without a specified timeframe and had not been informed that they might be randomly selected for a specific work-placement intervention, as per the cohort-randomised design (Consent form text: ‘I agree that I may subsequently be contacted by the research team to be offered an intervention that may help return to work, or an interview study about my experiences, but I understand that choosing to participate in these will be voluntary.’). Their accounts distinguished a wish to work or support research from willingness and capacity to take up this particular intervention at the time of invitation.

The situation is a bit complex right now… a job is not really on the agenda. (P08)

Case managers emphasised variability in expectations and understanding of study process, including unrealistic expectations, as key challenges, as well as the participants being very unpredictable in terms of scheduling and participation.

It really depends on what kind of participant you are dealing with. (CM5)

Many participants had very high ambitions. (CM4)

Some… do not quite understand why they have ended up here (study). (CM3)

The biggest challenge is in a way their unpredictability. So you set aside time, and then they are not there. (CM2)

This suggests a potential mismatch in how readiness is interpreted. Participants emphasise external constraints and timing and case managers emphasise internal factors (expectations, insight, motivation).

Mentions of suicidal ideation were raised by two case managers and three participants, highlighting the emotional strain that can be experienced within this population. Participants’ accounts linked these concerns to prolonged system-related frustration, lack of progress and perceived absence of support. For example, one participant described the situation as feeling that:

…either you take your own life, or you are strong enough to hang on until you are granted disability benefit (P09).

Case managers, in contrast, reflected on the challenges of recognising and responding to such distress, noting both the prevalence of unspoken suicidal ideation and a perceived lack of training to manage it appropriately. As one case manager stated,

How should I handle it really, in the moment, if there is a person with suicidal thoughts? It’s not uncommon in this group…and some of my participants were very close to this, without saying it out loud (CM1).

Neither case managers nor participants reported any specific adverse events, neither suicidal attempts nor physical injuries, to the trial management team in relation to the intervention participation.

#### Intervention structure and coordination

Participants experienced delays and poor coordination between components as frustrating. This corresponded with the recorded intervals between intervention steps, where the median time from the work familiarisation session to a placement offer was the longest in the pathway at 34 days (table 1).

It took a very long time from when you were invited to when you were to go out to the workplace. (P04)

Case managers and the placement recruiter similarly described fragmented delivery and unclear role boundaries, often having to define processes themselves.

I was somewhat missing clarity… I was a bit left to my own devices. (CM1)

Initially, I received quite a good briefing, a good explanation of what was wanted, but it did not really work in practice. So, they had a plan that in theory seemed quite good, but in practice it did not work. (PR)

Both groups identified structural and organisational weaknesses. This represents a system-level issue rather than a perception gap.

#### Work familiarisation session: reflection versus drift

Participants often reported experiencing the 2-day course as meaningful and reflective, especially the group aspect.

It triggered reflections… patterns that could be changed. (P02)

However, some noted dominance of system-related frustration, reducing focus.

The work familiarisation session facilitator observed discussions drifting towards complaints and helplessness.

System frustration… became a barrier… instead of being solution-focused. (WFS)

Participants sometimes found validation in shared frustration while the facilitator viewed it as potentially undermining outcomes.

#### Work placement: divergent experiences but shared challenges

Participants reported highly variable outcomes, with perceived benefits when placement fit well:

You are… part of a community. (P19)

Participants described disappointment and reduced motivation when they felt placements were poorly matched:

I was a bit disappointed with what I was offered. (P24)

Case managers, placement recruiter and trial coordinator emphasised matching difficulties due to: limited placement availability, unrealistic expectations and insufficient participant information.

It did not quite match what they thought they would get. (CM4)

Participants seem to experience mismatch as disappointment or harm while providers frame it as a structural labour market constraint.

#### Perceived outcomes: employment versus broader gains

Participants frequently highlighted non-work outcomes: self-understanding, acceptance and social belonging.

It is having something meaningful to do that is the most important. (P25)

Case managers similarly recognised mastery and mindset change as key outcomes:

Then there was someone who received a work placement that did not actually fit and what the participant had mentioned could… aggravate… the complaints. Or, aggravate the complaints. And not at all the kind of workplace the participant had envisaged. But thrived so well in the work placement, and was enormously grateful afterwards, that the participant had been given this opportunity. (CM4)

Both perspectives support a broader conceptualisation of outcomes beyond employment.

#### Summary of interview experiences and acceptability of the intervention

Taken together, participants and case managers describe a relationally driven intervention embedded within structural constraints, where perceived benefits depend on alignment between participant readiness, tailored support and realistic placement opportunities. While experiential accounts largely converge, differences in emphasis highlight underlying tensions between individual needs and system-level limitations.

## Discussion

### Summary of process evaluation primary findings

This process evaluation assessed implementation of the ReISE return-to-work intervention for unemployed individuals with persistent pain, examining reach, dose, fidelity, acceptability and contextual factors. Core intervention components were delivered with high documented fidelity among participants who engaged, particularly the work familiarisation session, and the intervention was broadly acceptable to those who participated. However, uptake was lower than anticipated, attrition was high and structural challenges related to the labour market, welfare system and intervention design constrained delivery and progression. These findings provide context for interpreting trial outcomes, consistent with process evaluation guidance that stresses the need to distinguish intervention theory from problems of reach, delivery, mechanism and context.^21^

Participants and staff described useful aspects of the intervention alongside difficulties in uptake, coordination and placement access. These findings identify conditions affecting delivery and engagement, but do not establish whether the intervention improves return to work or rule out limitations in its design. Supported work-placement models also depend on timely placement access, employer engagement and coordination with the welfare system.^11 34^ Some of the major challenges of conducting a trial on this target population are addressed below.

### Reach and recruitment of the target population

Recruitment relied primarily on social media self-referral, with minimal contribution from the Norwegian Labour and Welfare Administration or healthcare services. Although this approach generated a large initial sample, interview accounts indicated that willingness to join the research cohort did not necessarily imply willingness or capacity to undertake a work placement when invited. Wanting to return to work was an eligibility criterion, but no timeframe was specified and participants entered the cohort before being informed about random selection for the intervention. Similar challenges have been reported in unemployed chronic pain populations, where recruitment, retention and work readiness limit straightforward transfer of vocational models such as IPS.^34^ The cohort-randomised design may therefore have contributed to the high decline rate at intervention invitation (53%). The baseline comparison points in the same direction. Invitees who declined or could not be contacted did not differ from those who accepted in age, sex or time out of work, but rated their work ability and confidence in return to work lower and more often experienced a long time before returning to work. Although the differences were modest, non-uptake therefore appears more closely related to perceived capacity for work at the time of invitation than to demographic characteristics. These outcomes should be considered alongside the cohort characteristics: prolonged work absence, low work ability, low confidence in returning to work and complex pain-related and psychosocial barriers. These characteristics are associated with more complex return-to-work trajectories and may reduce readiness for intensive vocational intervention at a specific point in time.^11 35–37^ Women accounted for 87% of the ReISE cohort, compared with 59% of Norwegian work assessment allowance recipients with musculoskeletal diagnoses in December 2023 and 70% of disability-benefit recipients with musculoskeletal or connective tissue disease in December 2017.^38 39^ Differences in eligibility, diagnostic definitions, age coverage and reference years limit conclusions about representativeness.

### Interpretation of implementation components: fidelity and dose

Among participants who consented, key components were delivered with high documented fidelity, including consistent documentation of return-to-work plans and high adherence in the observed work familiarisation sessions. However, high fidelity to such structural components did not necessarily translate into effective delivery of the intervention’s relational and adaptive elements, which appeared more sensitive to staff experience, role clarity and contextual constraints. The small number of participants does not in itself indicate the capacity available for delivery. Case managers reported combining intervention work with clinical and/or teaching duties, with periods of low activity followed by several participants requiring support at once. Participant availability also varied. These accounts provide context for the scheduling delays, although the contribution of staff and participant availability to individual delays was not established.

Case managers reported that training provided a strong theoretical foundation but insufficient preparation for managing psychosocial complexity. This gap, documented in similar vocational rehabilitation process evaluations, highlights the need for practical training, supervision and clearer role boundaries.^40–42^ Implementation challenges, particularly delays in transitioning from work familiarisation session to placement, disrupted continuity. The median time to placement offer was 34 days, with substantial variability. These delays appeared to weaken participant engagement and momentum. In a comparable participatory programme for unemployed and sick-listed people with musculoskeletal disorders, delayed access to suitable workplaces was identified as the most operationally significant barrier, with a mean delay of 44.5 days to placement.^40^ Importantly, challenges in ReISE were concentrated at the interfaces between components rather than within them, indicating that coordination was the principal operational weakness. This is consistent with evidence that multidomain return-to-work interventions require integration of health-focused support, service coordination and workplace accommodation, rather than isolated delivery of single components.^13^

### Comparison with existing process evaluations

The challenges observed are consistent with prior process evaluations of return-to-work interventions. Studies have identified workload, limited organisational support, insufficient practice and delays between training and delivery as barriers, alongside difficulty in delivering relational components such as trust-building.^41 42^ Similar patterns are reported in IPS and participatory workplace intervention evaluations, where integration with health services, employer contact and delivery of workplace components were among the most difficult elements to realise in practice.^26^

Across studies, a consistent pattern emerged: multi-component interventions encounter the greatest difficulty at the boundaries between components, and relational delivery is harder to sustain than adherence to structure. The present findings indicate that delivery and engagement depend on coordination across clinical, workplace and welfare domains as well as on the individual components.

### Contextual and systemic barriers

Implementation was shaped by three interacting constraints: welfare system conditions, participant complexity and labour market limitations. First, the Norwegian Labour and Welfare Administration policies restricted referral pathways and employer access, while uncertainty about benefit consequences discouraged participation and progression. This aligns with prior qualitative work in the ReISE population, where benefit-related uncertainty and fragmented welfare support were described as barriers to return-to-work engagement, and with broader evidence that perceived work disincentives can undermine work participation.^43 44^

Second, the cohort exhibited substantial complexity, including prolonged work absence, low confidence, high fatigue, psychological distress and variable readiness. This indicates that meeting the eligibility criteria did not necessarily reflect capacity to enter an active placement pathway.^45–47^ Third, labour market conditions limited placement opportunities. The 6-week placement duration was often insufficient for employer engagement, and recruitment depended heavily on individual networks. These constraints operated cumulatively, limiting both entry into and progression through the intervention and highlighting the importance of intervention-context fit.^48^

### Candidate mechanisms and intervention assumptions

The qualitative findings showed that the case manager relationship to each individual participant appeared central to trust, validation and sustained engagement, while the work familiarisation session and placement could support mastery, structure and social re-engagement. However, these mechanisms depended on timing, continuity and placement fit. When delays occurred, or when placements did not match participants’ capacity or expectations, the intended pathway from preparation to work exposure was weakened, as found in similar studies.^49–51^ This suggests that these candidate mechanisms depended on several conditions, such as the readiness and motivation of the individuals included in the intervention, coordination between intervention stages and intervention staff, and credible workplace opportunities with meaningful work tasks, realistic working hours and demands matched to participants’ physical capacity.

### Practical implications and recommendations

Three priorities emerged for future delivery and evaluation of similar workplace interventions and/or trials. First, structured readiness assessment should identify the support needed to participate and guide timing, without becoming an additional exclusion criterion. It should be accompanied by clear information about what participation involves and its implications for benefits, and an opportunity to revisit a decision to decline. A stepped or stratified model may better match intervention intensity and timing to participant capacity.^52^ The qualitative accounts indicated differing support needs, but this evaluation did not identify discrete participant profiles or test whether they predicted uptake. Second, closer involvement of the Norwegian Labour and Welfare Administration, the public agency that administers sickness, disability, and unemployment benefits and brokers return-to-work support, could broaden recruitment beyond social-media self-referral. The latter reaches only those who encounter the study material and act on it, whereas the agency is already in contact with people receiving these benefits and could therefore better define and reach the eligible population. Two of the reasons given for declining, uncertainty about benefit consequences and poor timing in relation to other return-to-work measures, arise within the welfare system and can only be resolved there. Delivered as one of the agency’s own measures rather than as a separate study, the intervention would neither compete with obligations participants already had nor require a separate decision to enter research outside the agency. This would require agreement at agency level, with safeguards ensuring that participation remained voluntary and that declining carried no consequence for benefits. Third, delivery infrastructure should be strengthened through practice-based case manager training, supervision, protected time, clearer role boundaries and a more flexible placement design. Refining intervention content alone is unlikely to resolve these organisational and contextual barriers.

### Methodological strengths and limitations

To our knowledge, no process evaluation has been published of a return-to-work intervention for unemployed people with persistent pain: existing evaluations have concerned severe mental illness or sick-listed workers.^41 53^ Strengths include collecting data from participants and all intervention staff roles, which allowed triangulation across participants, case managers, the work familiarisation session facilitator, the placement recruiter and the trial coordinator.^54^ Inclusion of participants who declined the intervention is a strength, as it clarifies reach and acceptability beyond completers.^55^ Prospective quantitative process data and direct observation of work familiarisation sessions further strengthen validity.^56^ The evaluation and reporting of the findings were grounded in the MRC guidance and an explicit logic model, and interview data were coded by more than one researcher using the Framework method, supporting analytic transparency.^21 33^ Analyses were conducted and paper submitted for publication before the trial outcome data were available, reducing the risk that knowledge of effect estimates shaped interpretation (first submission date 30 June 2026—first results present to chief investigator estimated in September 2026).

Limitations include a predominantly female interview sample, limited direct observation of case manager delivery, potential recall bias and underrepresentation of some intervention stages in qualitative data. The comparison of invitees who accepted and those who did not was descriptive and was limited to baseline questionnaire measures. The inclusion of invitation decliners in the interviews broadens the accounts of non-participation but does not resolve these limits on assessing selection and representativeness. The predominantly female sample means accounts of acceptability and the candidate mechanisms may not transfer to men, who appear under-represented in this population. Because fidelity to the case-management component rested largely on documented Case Manager Forms rather than observed practice, the quality of delivery may be incompletely captured. The process evaluation was also conducted within a single trial setting, limiting transferability to systems with different welfare structures or employer incentives.^23 24^

## Conclusion

High fidelity was documented in the assessed components, and the ReISE intervention was acceptable to participants who engaged. However, low uptake and limited progression to placement restricted delivery of the full intervention to a small proportion of those invited. Deliverability across the wider eligible population therefore remains uncertain.

Further development should focus on support and timing adapted to participants’ circumstances, alongside improved access to suitable placements and closer coordination with welfare services. The trial outcomes should be interpreted in light of the limited intervention exposure and these delivery constraints.

## Supplementary material

10.1136/bmjopen-2026-125090 online supplemental file 1

10.1136/bmjopen-2026-125090 online supplemental file 2

## Data Availability

Data are available upon reasonable request.
