# Supplementary material for: Living with chronic pain and returning to work: lessons learnt from a process evaluation embedded in the ReISE trial
Source: BMJ Open. 2026 Oct 22;16(10):e125090. doi: 10.1136/bmjopen-2026-125090 (PMC13691893; doi:10.1136/bmjopen-2026-125090)
Supplement: Supplementary file 1 [file bmjopen-16-10-s003.pdf]

Returning people with persistent pain to work using Individual Supported Employment placements (ReISE)

Relevance to the call

The proposed project relates to the Welfare, Culture, and Society call, and the VAM programme. The project addresses the needs of a large vulnerable unemployed group with persistent pain. As the VAM programme outlines, a deeper understanding of large diagnosis groups comprising musculoskeletal disorders (which accounts for the majority of persistent pain) may make it possible to give such individuals a longer, healthier, and more rewarding working life. This in turn will help to safeguard welfare systems. Many with persistent pain may appear to have individually complex obstacles to working. However, a novel multidisciplinary approach, featuring matched work-placements and case management (tailored for people with pain) may offer a simple, practical, and effective solution. This intervention is designed to pave the way for rapid reintegration into the workforce and to equip people to enjoy many subsequent years of healthy and active employment, through enabling self-confidence and self-efficacy using evidence-based approaches. With international collaborative partners, and experts in chronic pain and work and health, the team has previously developed and established feasibility of this novel intervention in the UK. We now seek funding for a comparative study and cost-effectiveness analysis of the intervention compared to usual care, at scale, in Norway. Our non-research partners include a large national Norwegian recruitment company to provide placements and an umbrella organisation of prominent national health-profession and user-member societies that promotes strategies for tackling musculoskeletal pain. International globally-ranked academic collaborators, and central registry data outcome collection, will help to increase the capacity and quality of our research. Our intervention is robust to COVID-19 through design and technological reinforcement. A very strong user involvement has been integrated since the point of design and will continue throughout trial management, dissemination, and all our comprehensive planned impact activities. This will help to ensure that the intervention, trial, and dissemination of results will be relevant, have meaningful impact, and help to create new pathways to benefit users and wider society in the case of cost-effectiveness. These concepts closely align with key strategic objectives in the call and are emphasised as governing principles in the HELSEVEL, and VAM programmes. The programmes' ambitions are further supported through bringing together a multi-national group with diverse and relevant experience. In future, this group will be well-placed to respond to subsequent health-work calls, produce high-quality research, and contribute high-quality knowledge as output. Furthermore, with plans to supervise and mentor a PhD student and postdoc within this project, we support the wider programme objectives of building expertise and developing research competence in Norway. Over the longer-term, our aims are, through design, innovation and testing, to evaluate measures that enable an adaptable, inclusive and health-promoting working life, to increase the size and health of Norway's workforce.

1. Excellence

1.1 State of the art, knowledge needs and project objectives

Increasing work participation in sick and disabled populations improves health outcomes, reverses adverse effects of worklessness, reduces poverty, and improves quality of life and well-being.[1] Persistent (chronic) pain is the most frequent cause of sick leave and work disability in Norway; accounting for 11% and 9%, respectively.[2] Most chronic pain is of musculoskeletal (MSK) origin.[3] In 2019, around one-third of work days lost in Norway were due to MSK conditions.[4] There are gender differences in reporting, with 18% percent of men and 27% of women in Norway reporting MSK pain lasting six-months or longer.[5] In 2013, the costs of MSK conditions to the Norwegian health service were NOK 14.5 billion, and the total socio-economic costs were NOK 96 billion.[5] The average age of people reporting persistent pain in Norway is 48 (SD=16).[6] In people of this age, disability benefits increased between 2011 and 2020, and the percentage outside the labour market increased between 2018 and 2020 (in both cases by around 1.5%).[7, 8] However, these people potentially have many remaining working years to contribute.[9]

People with persistent pain want to do something useful. For many, a job is an essential part of self-identity; yet being out of work is too often their solution.[1] There is uncertainty about how best to help unemployed people with persistent pain return to work (RTW). Usual care for people with persistent pain is interdisciplinary in Norway, featuring pain management services supported by physicians, psychologists, physiotherapists and nurses.[10] Employment services take an individualised approach, and are flexible, including assessments of work ability, training, and vocational rehabilitation programmes involving

traineeships in sheltered businesses.[11] However, these services are not specifically tailored for people with pain. Effective vocational rehabilitation requires a combination of work-focused healthcare together with supportive workplaces; unfortunately, the two are generally not coordinated.[12]

Supported employment interventions, such as Individualised Placement and Support (IPS), are effective for helping people with mental health conditions to gain and retain employment.[13] Considerable interest has been generated in adapting this style of intervention to help people with persistent pain.[14-16] For example, in Oslo there is an ongoing study of the mental health IPS intervention for helping people with pain to RTW.[10] The mental health challenges that the original IPS packages were designed to tackle are frequently comorbid with persistent pain; either as a pre-existing condition, or as sequelae to psychosocial and physical challenges associated with living in pain.[17] People who are unemployed due to persistent MSK pain face different obstacles to working than those with mental health conditions *per se,* requiring work-place accommodations in the physical task environment.[18, 19] In contrast to IPS, where the user is largely passive and simply supported, engaging people with persistent pain in identifying their own individual (psychosocial) obstacles to work ability, agreeing on the actions to overcome those obstacles in a RTW Plan, and combining pain management advice, may be more effective for this group.

In 2020, we reported a nominal group study comprising international experts, who recommended that adaptations of supported employment interventions for persistent pain focus on facilitating integration/communication between systems and institutions, and emphasised a need to produce research for policy-maker consumption.[20] Additionally in 2020, we described the results of a feasibility study of an adapted supported employment placement intervention, run in the UK, which featured work placement provision with case manager (CM) support.[15] In this novel intervention, we trained case managers to act as a bridge between systems and institutions, engage people about obstacles to work, and collaboratively agree a RTW plan. We also provided supportive work placements. The developed intervention was acceptable and valued, with around 20% of the sample obtaining paid employment within the short six-month follow-up: self-perception of satisfactory functioning in role and management of identified obstacles, may be key to increasing confidence for this group. As the study focused on feasibility, the effectiveness and cost-effectiveness of the intervention are unknown. Without intervention, the probability of RTW after two years of unemployment is thought to be close to zero (*i.e*. the quasi-experimental rate estimate of 20% in our feasibility study appears to be promising in this context).[15]

RTW rates for traditional vocational rehabilitation in the unemployed (rather than sick-listed) pain population are unclear. In trials of interventions, outcome measures have often been expressed with employment success constituting as little as one day of paid work.[21] A focus on sustained RTW will be more useful and relevant.[22] Our aim is to establish the effectiveness and cost effectiveness of a supported work placement intervention with case management, for improving sustained RTW in people who are unemployed and who have persistent pain. Specifically, our objectives are to adapt our developed intervention for use with a Norwegian population; to pre-pilot adaptations; test recruitment; define pragmatic usual care; and then do a full-scale trial, identifying sustained RTW rates and several secondary outcomes, including health-related quality of life. The output will inform tailored policy advances and the management of inclusive health-promoting working life.

1.2 Research questions and hypotheses, theoretical approach and methodology

The project is divided into four work packages (WP) and a PhD studentship. WP-I to WP-III are scientific work packages that address the research questions below. WP-IV relates to dissemination and exploitation and is described in Section 2. PhD objectives are described in Section 3.

Research questions

WPI: Pre-pilot of adaptions, recruitment, and clarification of population-specific care in employment services

A) Can we adapt and deliver a novel supported work placement intervention that can be used in Norway for people of working age who are unemployed and have persistent pain?
B) What existing employment services does this specific population typically receive as usual care?
C) Can we adequately recruit to a cohort study and sub-sample an intervention group?
D) Are we able to recruit people receiving Arbeidsavklaringspenger (Work Assessment Allowance)?

WP-II: *Cohort randomised controlled approach*
What is the effectiveness and cost-effectiveness of a supported work placement intervention, compared to usual care, for improving sustained RTW and quality of life in unemployed people who have persistent pain?

WP-III: *Process evaluation*
What are the delivered processes in the package; in terms of fidelity, context, reach, dose, continuity, participation, recruitment, engagement, and referrals?

<u>Work Package I – Pre-pilot study of the ReISE intervention</u>

Description of the intervention

The ReISE intervention comprises training people with experience of working with pain patients.[15] We will train between two and four CMs (from occupational therapy/nursing/physiotherapy backgrounds) to identify obstacles to RTW using the Psychosocial Flags Framework and a set of stem questions.[23] This takes a biopsychosocial perspective to identify and tackle obstacles across personal, workplace and social domains, culminating in a RTW Plan. We will provide training for CMs on pain management strategies and benefits systems (since CMs will be unaffiliated with the Norwegian Labour and Welfare Administration (NAV), which our user input suggests may be important). The trained CM contacts participants by phone to make an initial assessment, explore work-aspirations and skills, and helps the participant to identify work obstacles. A face-to-face assessment is offered if the CM considers one needed. The CM then matches the participant with a placement, based on skills, aspirations, and suitability. To this end, we have partnered with Manpower, one of the leading staffing providers in Norway, with a network of employer partners. Our partner will provide six-week part-time work placements of up to 16-hours per week (for those who may be on *Arbeidsavklaringspenger* (AAP) and must work less than 50% FTE). Placements will span roles in administration, warehouse, transport, media and marketing, IT, sales and service, finance, accounting, and payroll. Placement provision will adhere to any adaptive working arrangements as may be required at the time with respect to COVID-19. Once a placement is arranged, the CM, the participant, and supervisor meet (physically/virtually) to agree a RTW plan and discuss whether accommodations are needed to overcome identified obstacles. Our support model features an initial familiarisation period to help embed the person into their role and discuss work-focussed pain management. The session will be run over two-days in required geographic locations, and possibly by video link (which we will explore in our pre-pilot), and delivered in conjunction with the Competence Centre for Inclusive Working Life (KAI), at OsloMet. KAI has an extensive track record of research in this area. With KAI, the INTEGRATE Pathways to Work team, and the Norwegian Council of Musculoskeletal Health (RMH; a user-facing umbrella organisation with both health and user member groups) coordinating user group input (*cf*. Section 3), we will culturally adapt existing familiarisation material. Adapted materials will be available in Norwegian and English, following success of offering these languages in a trial of motivational interviewing we are conducting for those sick-listed with pain in Norway.[24] The CM then provides regular telephone/video support to the participant and supervisor, which is known to be effective.[25] If needed, the CM may make referrals to existing health services – for example, for CBT and physiotherapy, which are effective.[19] The CM also aids clinicians in providing work-focused health-care through provision of existing evidence-based resources. Similar materials are given to all participants and placement providers. Examples include elements of the Health-Work Toolbox, and information/advice leaflets on the work-health interface. [26-28] We will adapt and translate these materials into Norwegian (with permissions), and we have obtained costs from the publisher for translation and typesetting. In the case of more challenging and persistent obstacles, the CM may refer to work-focused counselling services, for example at one of Norway's Centres for Job Coping (PES).

Pre-pilot study, target population, and recruitment

There is a small risk that unknown circumstances could threaten feasibility of a full trial in Norway (*e.g.* through inadequate recruitment or unacceptability to participants). Therefore, we will conduct a pre-pilot study to test recruitment pathways, adaptations, and confirm we can deliver the intervention. We will work with Manpower to recruit nationally (although we anticipate most recruitment around Oslo) people aged between 16-64, who have been out of work for at least one-month, and had pain for more than three-months that interferes with work ability. We will exclude those aged 65 or over as their future time in work would be relatively limited. We will make no exclusions for comorbidities unless these would prevent RTW.

We will exclude people insufficiently fluent in Norwegian or English to give consent, which approximates to the fluency required to enter the open labour market in Norway. Manpower has an existing relationship with the Norwegian Labour and Welfare Administration (NAV) and direct links with 17 NAV offices who actively refer for work experience schemes. Manpower will do direct recruitment and engage NAV offices. We will also mount an independent advertising campaign across social media, pain groups, and charities (including pain and rheumatological charities: *Ryggforeningen, Norsk Revmatikerforbund, foreningen for kroniske smerter*), public facilities, job clubs, and *Frisklivssentralene* (*i.e.* 'healthy living' centres, which provide help with health and work matters). We know from our feasibility study that recruitment from pain clinics is not efficient. We will confirm inclusion criteria, and that we can recruit a pilot test cohort (rationale below) of 66 people, sub-sampling 17, within a six-month time frame. We will offer NOK 200 of high street vouchers to those joining the cohort to help recruitment and engagement. We will examine the extent to which we are able to recruit those on AAP benefit (see above) and those receiving an alternative permanent disability benefit called '*Uføretrygd*', which has less stringent work rules. Results will refine our recruitment targeting for a full trial. We will explore remote CM support and work familiarisation by video link. If feasible, this will enable more diverse national recruitment in places where it may be impractical to send CMs/trainers. We will confirm intervention acceptability through focus groups with participants and interviews with placement managers. If any findings leave us unable to proceed to a main trial, the trial budget line will be returned to the funder and findings and lessons learned will be published.

Work Package II - Main trial

We will use a cohort randomised controlled approach.[29] This involves sampling a cohort of people and obtaining consent from everyone. We will then randomly sample a sub-group to whom we will offer the intervention and obtain consent for trial participation. This approach is useful where obtaining consent to be randomised not to receive an intervention may be difficult, or if there is likely to be contamination of the test intervention. Moreover, it avoids ethical issues of non-consent that can be present in other designs performed for similar reasons (*e.g.* those associated with Zelen's design). By using 1:4 random sampling, we can almost half the number needed to receive the intervention. This will optimise information gained at a decreased cost, offering excellent value for money. To detect a difference of 20% in cumulative incidence of sustained RTW between the intervention group and those receiving usual care, assuming a usual care RTW rate as high as 10%, 80% power, and a 5% significance level, we will need data on 205 participants (164 control and 41 intervention).[15] Allowing 20% loss to follow-up, we will recruit 257 people. Initially, all will be recruited to an observational cohort study of the impact of being out of work with persistent pain. We will then randomly select one-in-four to be offered the intervention. At baseline, we will collect demographic data and use Norwegian versions of the following validated questionnaires: health-related quality of life (EQ-5D-5L),[30] SF-12,[31] the Hospital Anxiety and Depression Scale,[32] Dimensions of Work Ability,[33] the Warwick-Edinburgh Mental Well-being Scale,[34] PROMIS fatigue, and pain interference items (4a),[35, 36] and pain intensity.[37] We will collect follow-up data at three, six, and 12-months from randomisation, and: details of care received and services used (whole cohort); satisfaction with placement (trial group) or usual care/services (cohort); and from the trial group: work-role functioning;[38] psychosocial aspects of work;[39] RTW self-efficacy;[40] adaptations due to COVID-19; whether work makes pain worse; job offers; adverse events; healthcare referrals; and sustained RTW. For our primary outcome of sustained RTW, we will use registry data from NAV. A cost-line for this service is included in our budget. We define sustained RTW as the first continuous four-week period of 50-100% RTW.[22] We will analyse between-group differences in sustained RTW using a generalised mixed-effect model with logit link function, adjusting for age, gender, and area of pain, and fitting a random effect in CM allocation. We will fit linear mixed-effect models to estimate between-group differences in continuous secondary outcomes, and a zero-inflated Poisson model for differences in the number of job offers. An economic evaluation will be integrated into the design. This will be conducted from the personal social services (PSS) perspective.[41] At three, six and 12 months post-randomisation, we will also use registry data to estimate hospital and community health and social care resource use. Current Norwegian unit costs will be applied to value total resource use in both groups. Responses to the EQ-5D-5L and SF-12 will be converted into multi-attribute Norwegian utility scores.[42, 43] Results will be presented using incremental cost-effectiveness ratios (ICERs) and cost-effectiveness acceptability curves (CEACs). This accommodates sampling uncertainty and varying levels of willingness to pay for an additional QALY. We will consider constructing a decision-analytical model if one is needed.

Work Package III – Process evaluation

We will run a process evaluation parallel to the intervention in WPI-II. We will explore implementation, continuity of the provided package, reasons for participating and declining participation, and mechanisms behind cases of sustained RTW. Within these objectives, we will quality assure and assess recruitment; context (location, demographics, and circumstances); reach (*i.e*. proportion of eligible participants completing the study, and participants' characteristics); doses delivered/received; maintenance (*i.e.* numbers and reasons for participant drop-out and procedures used to facilitate continued participation); implementation (*i.e.* fidelity to the conceptual model and the extent to which the familiarisation sessions and intervention were implemented as designed); reasons for health referrals; and intervention acceptability and adherence.[44] To do this we will use observation techniques, data from field notes, interviews, focus groups with participants, CMs, and placement managers, diary records kept by CMs, and registry data. Interview and focus group participants will be purposively sampled for age, gender, area of work, and pain type. We will analyse data using a mixed-methods approach, with quantitative data being summarised descriptively, and qualitative data (from the focus groups and interviews) transcribed verbatim and analysed using the Framework method.[45] This involves researchers independently coding transcripts, creating a framework of themes, and analysing matrices of themes to identify patterns and interrelations between and within cases, as a function of characteristics and outcomes (*e.g.* achieving sustained employment).[45]

1.3 Novelty and ambition

The ambition and novelty of our proposal comes from expanding one aspect of health-work science to solve a parallel problem. We have taken an approach known to help people with mental health difficulties to RTW and adapted it to tackle persistent pain. We go beyond the state of the art through the inclusion of novel components that are more conceptually appropriate for this group, and more likely to effectively solve this problem. We provide individually matched supportive work placements, after first actively engage people in identifying their own individual (psychosocial) obstacles to work, forming a plan, and providing advice on pain management. This is in contrast the traditional IPS approach, where the user is largely passive, and simply supported, the aim is to provide paid work straight away, but where health aspects are not first addressed. We have already established feasibility and acceptability of this intervention and its novel features. Our aim now is to adapt for delivery in Norway and establish effectiveness and cost-effectiveness. In Norwegian, the acronym word '*Reise*' means travel, or journey. Using our interdisciplinary approach, working with a large leading recruitment company and a user-facing national council, we will apply evidence on navigating the work-heath interface to enable the journey to work participation for people in pain. The study will provide valuable knowledge about how best to help to this population and inform tailored policy.

2 Impact

2.1 Potential impact of the proposed research

There are clear pathways for multiple impact vectors from this work. Our study will provide valuable information on effectiveness and cost-effectiveness of an intervention that empowers participants to identify their own obstacles to work, and provides a safe environment to overcome those obstacles with support. A cost-effective intervention will have several direct societal impacts. Importantly, it will provide a pathway back to work for an experienced group at a time when the percentage of older people outside the workforce has increased and is unsustainable. This will lead to more years of work, more opportunities, and create value for industry. Working longer will not only decrease the period at the end of working life, reducing demand on welfare services, but create synergy through simultaneously improving health and lowering demand on health services. The intervention is less labour intensive (and thus less expensive) than IPS approaches that seek to find paid work with time-unlimited support. As the ReISE intervention provides work-focused and psychosocial support and practically addresses work obstacles, it is conceptually more appropriate for the group and may provide greater health impacts. Knowledge of how cost-effectiveness compares to usual care will impact policy makers and researchers' directions of travel, regardless of outcome.

Family and partners often have care duties for those with persistent pain and relationship strains impact quality of life.[46, 47] Improving work participation would thus improve quality of life not only for people with pain, but also for partners and families through reducing these strains. There will be benefits that result from enabling workplace mixing within and between generations. Participants in our feasibility

study commented that the intervention helped them feel more connected and less alone.[15] Thus the intervention may not only reduce the national social burden of disability, but also facilitate integration and good society. The knowledge created will address several UN sustainable development goals. In particular, UN sustainable development targets 8.5 (full employment for men and women) and 10.2 (socioeconomic inclusion); the intervention is designed to help both women (more of whom will join the intervention given the greater prevalence)[15] and men with disabilities achieve full productive employment. In the case of a cost-effectiveness, time to widescale national impact would be possible within five-to-eight years, following adoption and roll-out of the intervention. To this end, we attach importance to ensuring that outputs will be written for policy-makers and users. We aim to inform and influence national policy within the short-to-medium term (*e.g.* between -1 and five years of study completion). Finally, as materials from our intervention will be adapted and translated in the earliest phase of the research, these will be available for use prior to the end of the study. The activities described in WP-IV that follows, will raise the profile of the resources providing a pathway to medium-term impact for people with persistent pain.

2.2 Measures for communication and exploitation

Work Package IV – Dissemination and Exploitation

To maximise impact and use of findings, we have established a comprehensive communication and exploitation plan across four target areas/groups (Table 1):

I. scientific, health, and education communities (informing research, practice, and teaching);
II. people with persistent pain (who will benefit from the scheme and/or adapted materials);
III. NAV (who will benefit from knowledge about what works, when, and for whom); and
IV. policy makers (for whom results will inform tailored policy making).

The applicants have a strong track record of publishing in high-impact journals. We will publish four articles in high-impact journals. Articles will comprise a protocol; a report on the internal pilot study; the main trial and cohort results paper; and a process evaluation. We will target a journal with a very high impact factor for the trial report, such as the BMJ or Lancet, in which members of our group have multiple publications. We will include user authors to ensure lay summaries and abstracts are accessible and user-relevant. We will present results at national and international conferences. A PhD dissertation will describe the influence of demographics on joining, mechanisms leading to sustained work participation, predictors of sustained RTW, and hospital, community and social care resource use in the trial and cohort samples.

Table 1 – Dissemination and exploitation package overview

Target groups	Relevant output	Dissemination means	Exploitation
I. Scientific, health, and education communities	Pre-pilot results; effectiveness and cost-effectiveness estimates; and process evaluation.	Four open-access publications; conference presentations; PhD; teaching materials and RMH report dissemination and seminars.	Improving of RTW management, intervention design; education; and generating new research and collaborations.
II. People with chronic pain	Case manager support; opportunity to RTW and improve income and QoL; provision of translated support materials to help pain management in the workplace.	User-designed website with support materials; social media signposting; broadcasting; popular science; and lay report for RMH dissemination and for those involved.	Improving RTW and QoL via trial and national schemes (if cost-effective) and user-facing supportive materials, and help-point signposting.
III. NAV	Demographic model of those most likely to benefit; executive summaries; and resources to facilitate training/changes to implement working practices.	Seminars and consultation functions; a detailed manual documenting the intervention; and translated training resources.	Implementation of schemes, with identification and targeting of those individuals most likely to benefit.
IV. Policy makers	Details of intervention effectiveness and cost, provision and fidelity for reproduction; information on people's concerns relating to policy and their circumstances and willingness to RTW.	Policy briefs with dedicated web platform – *e.g.* www.goodworkgoodhealth.com Bespoke seminars and consultation functions.	Creation of new health management policy with interactions between national schemes for helping this group, and benefits through decreasing service use.

For users, we will do national broadcasting interviews and write popular science articles to disseminate results and raise the profile of the intervention and its advice materials. In addition to representing the societal problem from a user perspective, and enhancing design and user involvement throughout the programme, RMH promotes strategies for managing MSK pain. RMH will help raise the profile of the intervention through disseminating reports and arranging seminars for its member organisations. We will create lay reports for the consumption of all participants and users. We will engage people with persistent pain directly via a bespoke website and dedicated social media channels, which will be updated at least weekly by our trial manager to promote resource updates, debates, and relevant new articles and policy. We will actively engage those most likely to use our resources in helping to create them; involving RMH and our user group in material adaptation and website design. We will engage NAV and policymakers through seminars and consultation dialogues; creating executive summaries for every NAV office in Norway. Implementation of our intervention could require changes in working practices within NAV when working with this group. Presently, staff are trained to a specification similar to IPS, in as far as schemes tend to follow a place-then-train approach without coordinating work-focused healthcare. We aim to offset this by providing training resources to facilitate any necessary changes. We will also a create a Norwegian language manual documenting processes, fidelity and results in detail, making this available in addition to an executive summary. We will engage policy makers directly and supply tailored policy briefs, which we have previously used to inform policy-making in the UK on a platform which our policy lead has helped implement.

3 Implementation

3.1 Project manager and group collaboration

We have a strong international team. The project will be led by Froud (Kristiania University College), who previously led the feasibility study of this intervention in the UK (funded by Versus Arthritis (formerly Arthritis Research UK)). Froud has worked for NIHR Research Design Services in the UK, held senior academic posts at clinical trials units (Barts and the London School of Medicine, and Warwick Medical School), and has a track record of leading research. Froud will have the support of an expert team. Professor Burton (Subcontractor) is an expert advisor to the UK Department for Work and Pensions and has an OBE for services to occupational healthcare. He has authored seminal papers and guidance used in work and health, and was instrumentally involved in the design and set-up of the UK's 'Fit for Work' service. Professor Underwood (University of Warwick) is an NIHR Senior Investigator, with over 250 original peer reviewed publications, largely in musculoskeletal disorders/chronic pain. He is chief or co-investigator on current grants valued at >£10M. He was, until 2017, Director of the highly successful Warwick Clinical Trials Unit. On behalf of National Institute of Health and Social Care Excellence (NICE) he has chaired two NICE guideline development groups (Back Pain 2009, Headache 2012). He was part of guideline development group for osteoarthritis research society international (OARSI) guidelines for conservative management of osteoarthritis (2014 & 2019). Burton, Underwood, and Froud have all advised the UK's Department for Work and Pensions on work and health. Professor Grotle (OsloMet) is an experienced MSK researcher who has led research in measurement, worked with FORMI, and Oslo University Hospital. She currently has a grant of approximately NOK 11M to research the use and cost-effectiveness of Motivational Interviewing by NAV-employees, and a Stratified Vocational Advisory Intervention, provided by trained physical therapists to sick-listed people with MSK pain (the MI-NAV project). Småstuen is a biostatistician at the Faculty of Health Science (OsloMet). She has more than 200 publications in high ranking journals (including JAMA and the Lancet) and experience with conducting RCTs and large registry-based studies. Professor Kisa (Kristiania University College) is a health economist who has worked with the World Health Organisation on studies including Innovative Care for Chronic Conditions, and the Global Burden of Disease. He was principal investigator of WHO WHS-Turkey and of the National Burden of Disease and Cost-Effectiveness of Essential Health Services in Turkey. Malmberg-Heimonen (Work Research Institute, OsloMet) is affiliated with KAI and INTEGRATE Pathways to work research groups. She is an experienced trialist in the areas of social research and work and health. The INTEGRATE research group (which is funded by the Research Council of Norway) is focused on improving coordination between health, labour and work services; a core concept of ReISE. *Student* (name blinded) is the PhD student we have selected for this project. *Student* has excellent organisational skills, and demonstrated effective ability to manage projects and publish research. *Student* excelled on an MSc programme in Pain Management at University of Edinburgh, winning the prize for Best Student two years in

a row. Malmberg-Heimonen will be the secondary PhD supervisor with Froud as the primary. The primary objectives of the PhD work will be to examine the influence of demographics on joining the intervention, to study the mechanisms leading to sustained work participation, and also to predictors of sustained RTW within the trial arm (*i.e.* within secondary analyses). While *Student* will be most involved in WPI and WPIII, the student will also make a contribution to the overall running of the trial as appropriate, and with economist supervision, assist on estimation of hospital and community health and social care resource use, and be involved in the process of culturally adapting existing materials for use in the trial in WPI. Lunestad, our user representative, is a fully participating user co-applicant who also coordinates the FORMI user group network, and understands challenges with respect to working with persistent pain.

We have strong non-research partners for this project that represent the societal problem from perspectives of industry, people with pain, and health groups. Founded in 1965, Manpower is a large staffing provider in Norway providing employment services to a wide range of sectors. Manpower has over 6,000 workers in daily employment with a strong track record of recruits (66%) to permanent positions. Manpower is an ideal partner to support this research and its national recruitment ambition. RMH, is an umbrella organisation for 17 prominent national patient organisations, health professions, medical speciality associations, as well as the Norwegian Directorate of Health. RMH is well-placed to promote evidence-based strategies within these organisations as well as for people with pain. RMH is patient-oriented and patient-facing. During design, and throughout management RMH will help represent the societal problem from a user perspective. The complementary nature of the combined team is evidenced by the good fit of non-research partners and the academic expertise and track record of internationally ranked publications in relevant specialist areas. The group comprises experts in work health policy (consultancy from Burton), trials (Underwood, Malmberg-Heimonen), statistics and methodology (Småstuen/Froud), health economics (Kisa), and pain research (Grotle, Underwood, Burton, Froud). The partnerships of KAI, INTEGRATE (OsloMet), RMH and Manpower, provide additional expertise relevant to work and health in Norway. Our international partners bring leading expertise and a very strong track record in pain trials (*e.g.* COPERS, PLoS Medicine, 2016; 13(6); BeST, Lancet, 2010, 375(9718); I-WOTCH, BMJ Open, 2019, 9(8); CHESS, BMJ Open, 2020, 10(4); UK BEAM, BMJ, 2004, 329:1377). For example, the UK BEAM trial is the largest trial of manual therapy for back pain ever conducted, and informed both European and NICE guidelines on treatment and management.

3.2 Project organisation and management

Facilities

Kristiania University College (KUC; est. 1914) has enjoyed rapid growth and development over the past three-years, with grant income increasing by ~260%. KUC is housed in a NOK 500M state of the art premises at Kirkgate in downtown Oslo, which meets the highest environmental standards underpinning a strong commitment to reducing the carbon footprint of research. The school has excellent library facilities, both electronic and physical. Research is conducted within appropriate legal and ethical frameworks, supported by standard operating procedures (SOPs) and data management policies. The institution will provide research governance support, quality assurance, Good Clinical Practice training, dedicated servers for encrypted data, and administrative, programming and database support. University of Warwick is ranked within the top 100 universities in the world. Its trials unit has a large portfolio addressing common chronic MSK disorders. Five relevant trials (BeST, MINT, OPERA - HTA ref 06-02-2001, SARAH, PRE-Fit, COPERS - NIHR ref RP-PG-0707-10189) were funded to a value of approximately £9M. OsloMet's Centre for Work Inclusion (KAI), was established in partnership with the Norwegian Labour and Welfare Service (NAV), in 2017. The centre's goal is to produce and disseminate knowledge to increase the employment opportunities of groups outside, or on the margins of, the labour market. KAI delivers important research and research (or literature) reviews and disseminates knowledge to the public, policy makers, professions, and the public sector and affiliated services. OsloMet, which currently hosts around 120 PhD students, has the infrastructure and necessary experience to support the PhD student and is able to award the PhD. Manpower has 33 ISO9001 certified offices across Norway, with 13 around Oslo, where we anticipate most recruitment. RMH offers the facility of its large network of user-groups and professional organisations.

Timescales

The project will run over 36-months (Figure 1), submitting our ethics application prior to the project start date. We will recruit and train staff, register, and submit a protocol for the trial prior to recruitment.

Including the pilot, the delivery phase for the six-week work placements will begin in Q3 and run for 18-months. The process evaluation will be completed in Q9. The final follow-up collection and writing of the main trial paper for submission will be completed in the final quarter, at which point the PhD thesis will be submitted. Dissemination and impact activities in WP-IV will continue beyond the life of the project.

Co-applicant, staff activities, and project management

Work Package I – Pre-pilot study. Leader: Underwood and Malmberg-Heimonen. Collaborators: Warwick, Manpower, RMH, KAI, and OsloMet. The pre-pilot will be co-led by Underwood (0.05 FTE) and Malmberg-Heimonen (0.05 FTE). We will recruit a postdoc (0.67 FTE) to manage research activities and contribute to report writing, and a trial manager (TM; 0.75 FTE) to manage ethical, financial, contractual matters, and to monitor progress and tasks. We have found a dedicated TM helps with meeting targets and milestones, as provides a consistent first point of contact for participants, staff and collaborators. The PhD student (1.00 FTE) and postdoc will run interviews and focus groups to explore intervention acceptability. We will appoint an independent data monitoring committee (DMC) to monitor this phase (and WP-II), as well as an independent steering group (SG) to judge criteria for proceeding to WP-II. Adaptation of materials will be done in conjunction with RMH, KAI and INTEGRATE, with input from Burton. In addition to providing placements, Manpower (0.70 FTEs; 0.50 in-kind) will train a recruitment consultant and administrator to manage recruitment and placement options through pilot and trial phases. RMH (0.10 FTE; 0.05 in-kind) will provide a co-applicant for management input and an administrator to coordinate user input.

**Key activities and milestones**	**2021**	**2022**				**2023**				**2024**		
Quarter	*4*	*1*	*2*	*3*	*4*	*1*	*2*	*3*	*4*	*1*	*2*	*3*
Ethics approval, staff recruitment, training, and protocol publication.	X	X										
User, TMG, DMC and SG meetings	X	X	X	X	X	X	X	X	X	X	X	X
**Work Package I** – translation and adaptation of materials, recruitment and intervention delivery	X	X	X	X	X							
**Work Package II -** recruitment and intervention				X	X	X	X	X				
Data collection at 0, 3, 6, and 12 months			X	X	X	X	X	X	X	X	X	
Statistical plan, analysis of data and WP-II paper submission		X		X							X	X
**Work Package III -** Process evaluation, analysis and submission of paper					X	X	X	X	X			
Writing of thesis and PhD submission	X	X	X	X	X	X	X	X	X	X	X	X
**Work Package IV** – Dissemination and exploitation (to surpass study time period)						X	X	X	X	X	X	X

Figure 1: Gantt chart of activities and project milestones, by year and quarter. Grey shading indicates preliminary work, and green indicates an activity or milestone and blue represents paper submission as part of WP-IV

Work Package II – Main trial. Leader: Froud. Collaborators: Warwick, Manpower, RMH, KAI, and OsloMet. RF will take overall lead and lead WP-II (0.20 FTE). The TM will ensure activities are run in-line with SOPs, and enter data if required (with PhD/postdoc validation). *Student* will perform some research activities with the postdoc and senior oversight, and use registry data (with economist supervision) to estimate hospital, and community health and social care resource use. Monthly trial management group (TMG) meetings, and quarterly DMC and steering group meetings will be held. Småstuen will prepare a statistical analysis plan and conduct analyses (0.05 FTE). We will recruit two case managers (0.20 FTE in the trial period). We will subcontract Burton (0.05 FTE) to deliver training to case managers and facilitators (who we will also recruit (0.15 FTE)), who will run sessions for small groups (*e.g.* 4 to 7) of people recruited to the intervention.

***Work Package III – Process Evaluation. Leader: Grotle. Collaborators: Warwick, Manpower, RMH, KAI, and OsloMet*.** Grotle (0.05 FTE) will lead the process evaluation, with *Student* doing interviews and user-involvement management, and recruiting participants for WP-III with postdoc assistance. *Student* will act as interviewer/facilitator (following training), analyse qualitative data, and conduct process evaluation interviews and their analyses, leading the writing of this paper for publication.

***Work Package IV – Dissemination and exploitation. Leader: Froud.* Collaborators: All.** Burton's advice will help ensure output has a strong policy relevance. The TM will manage website and social media with the aid of RMH and users; first drafts of all reports and papers will be written by either Froud (WP-II), the postdoc, or *Student* (WP-I, III, and IV) with subsequent group collaboration and disseminated in-line with our plan.

User involvement

Good user involvement is essential to relevance and flows through all aspects of this project; from design of the original intervention, generation of the current research questions and collaborative design of associated methods, through to project management, results interpretation, and dissemination. All senior investigators have experience in forming and working with user groups, and RMH will facilitate user group meetings. We have included a user representative (associated with the FORMI user group network) on the design of this proposal, who has been instrumental in shaping development. Lunestad is a fully participating member of our team and will be a co-author on all reports, as is our usual practice. Using the FORMI user group service (which is actually coordinated by Lunestad), we will work with RMH to establish a wider lay reference group of five to seven people. We will consult this group over the lifetime of the study and key dissemination, engaging those likely to use project materials and outputs in helping to create them.

References

1. Waddell G, Burton AK. Is work good for your health and well-being? Department for Work and Pensions, 2006.
2. Laerum E, Brage S, Ihlebaek C, Natvig B, Aas E. Et muskel- og skjelettregnskap: forekomst og kostnader knyttet til skader, sykdommer og plager i muskel- og skjelettsystemet: MST-rapport. Oslo: Muskel og Skjelett Tiåret, 2013.
3. Elliott AM, Smith BH, Penny KI, Smith WC, Chambers WA. The epidemiology of chronic pain in the community. Lancet. 1999 Oct 9;354(9186):1248-52. PMID: 10520633.
4. NAV. Fortsatt stabilt sykefravær i Oslo 2019 [cited 2020 May 27]; Available from: https://www.nav.no/no/lokalt/oslo/pressemeldinger/fortsatt-lavest-sykefravaer-i-oslo.
5. Kinge J, Knudsen; A, Skirbekk V, Vollset S. Musculoskeletal disorders in Norway: prevalence of chronicity and use of primary and specialist health care services. BMC Musculoskeletal Disorders. 2015;16(75).
6. Rustoen T, Wahl AK, Hanestad BR, Lerdal A, Paul S, Miaskowski C. Gender differences in chronic pain--findings from a population-based study of Norwegian adults. Pain Manag Nurs. 2004 Sep;5(3):105-17.
7. SSB. Labour force survey. Oslo: Statistics Norway, 2020.
8. NAV. Disability benefit - Quarterly statistics. 2020 [Aug 11]; Available from: https://www.nav.no/no/nav-og-samfunn/statistikk/aap-nedsatt-arbeidsevne-og-uforetrygd-statistikk/uforetrygd.
9. UNECE National Report on Ageing 2016 – NORWAY. Follow-up to the Regional Implementation Strategy of the Madrid International Action Plan on Ageing. Oslo, Norway: Norwegian Ministry of Labour and Social Affairs., 2016.
10. Linnemorken LTB, Sveinsdottir V, Knutzen T, Rodevand L, Hernaes KH, Reme SE. Protocol for the Individual Placement and Support (IPS) in Pain Trial: A randomized controlled trial investigating the effectiveness of IPS for patients with chronic pain. BMC Musculoskelet Disord. 2018 Feb 13;19(1):47. PMID: 29433493. doi: 10.1186/s12891-018-1962-5.
11. Sveinsdottir V, Tveito TH, Bond GR, Grasdal AL, Lie SA, Reme SE. Protocol for the SEED-trial: Supported Employment and preventing Early Disability. BMC Public Health. 2016 Jul 15;16:579. PMID: 27422271. doi: 10.1186/s12889-016-3280-x.
12. Waddell G, Burton AK, Kendall N. Vocational rehabilitation – what works, for whom, and when? : TSO; 2013.
13. Brinchmann B, Widding-Havneraas T, Modini M, Rinaldi M, Moe CF, McDaid D, Park AL, Killackey E, Harvey SB, Mykletun A. A meta-regression of the impact of policy on the efficacy of individual placement and support. Acta Psychiatr Scand. 2019 Nov 16. PMID: 31733146. doi: 10.1111/acps.13129.
14. Rodevand L, Ljosaa TM, Granan LP, Knutzen T, Jacobsen HB, Reme SE. A pilot study of the individual placement and support model for patients with chronic pain. BMC Musculoskelet Disord. 2017;18(1):550.
15. Froud R, Grant M, Burton K, Foss J, Ellard DR, Seers K, Smith D, Barillec M, Patel S, Haywood K, Underwood M. Development and feasibility of an intervention featuring individual supported work placements to aid return to work for unemployed people living with chronic pain. Pilot Feasibility Stud. 2020;6:49.
16. Linaker C, Fraser S, Price C, Maguire N, Little P, Madan I, Pinedo-Villanueva R, Coggon D, Cooper C, Ntani G, Walker-Bone K. Individualised placement and support programme for people unemployed because of chronic pain: a feasibility study and the InSTEP pilot RCT. Health Technol Assess. 2021 Jan;25(5):1-72. PMID: 33501902. doi: 10.3310/hta25050.
17. Turk DC, Rudy TE. Toward an empirically derived taxonomy of chronic pain patients: integration of psychological assessment data. Journal of consulting and clinical psychology. 1988 Apr;56(2):233-8. PMID: 3372831.

18. Patel S, Greasley K, Watson PJ. Barriers to rehabilitation and return to work for unemployed chronic pain patients: a qualitative study. European journal of pain. 2007 Nov;11(8):831-40. PMID: 17320437. doi: 10.1016/j.ejpain.2006.12.011.
19. Waddell G, Burton AK, Kendall N. Vocational rehabilitation – what works, for whom, and when? . Vocational Rehabilitation Task Group, 2013.
20. Froud R, Amundsen P, Bartys S, Battie M, Burton K, Foster N, Johnsen T, Pincus T, Renemani M, Smeets R, Sveinsdottirg V, Wynne-Jones G, Underwood M. Opportunities and challenges around adapting supported employment interventions for people with chronic low back pain: modified nominal group technique Disability and Rehabilitation. 2020;3:1-8.
21. Bejerholm U, Areberg C, Hofgren C, Sandlund M, Rinaldi M. Individual placement and support in Sweden - a randomized controlled trial. Nord J Psychiatry. 2015 Jan;69(1):57-66. PMID: 24983382. doi: 10.3109/08039488.2014.929739.
22. Jensen C, Jensen OK, Nielsen CV. Sustainability of return to work in sick-listed employees with low-back pain. Two-year follow-up in a randomized clinical trial comparing multidisciplinary and brief intervention. BMC Musculoskelet Disord. 2012 Aug 25;13:156. PMID: 22920158. doi: 10.1186/1471-2474-13-156.
23. Kendall N, Burton AK, Main C, Watson P. Tackling musculoskeletal problems - A guide for the clinic and workplace. Identifying obstacles using the psychosocial flags framework. The Stationary Office2009.
24. Oiestad BE, Aanesen F, Lochting I, Storheim K, Tingulstad A, Rysstad TL, Smastuen MC, Tveter AT, Sowden G, Wynne-Jones G, Fors EA, van Tulder M, Berg RC, Foster NE, Grotle M. Study protocol for a randomized controlled trial of the effectiveness of adding motivational interviewing or stratified vocational advice intervention to usual case management on return to work for people with musculoskeletal disorders. The MI-NAV study. BMC Musculoskelet Disord. 2020 Jul 28;21(1):496. PMID: 32723318. doi: 10.1186/s12891-020-03475-z.
25. Burton AK, Kendall N, McCluskey S, Dibben P. Telephonic support to facilitate return to work: what works, how, and when? . Department for Work and Pensions, 2013.
26. Burton AK, Waddell G. Advising Patients About Work: An Evidence-based Approach for General Practitioners and Other Healthcare Professionals: The Stationary Office; 2008.
27. Burton AK, Waddell G. Health and Work - Employee's booklet: The Stationary Office; 2007.
28. Waddell G, Burton AK. Work and Health: Changing How We Think About Common Health Problems: The Stationary Office; 2007.
29. Relton C, Torgerson D, O'Cathain A, Nicholl J. Rethinking pragmatic randomised controlled trials: introducing the "cohort multiple randomised controlled trial" design. BMJ. 2010 Mar 19;340:c1066. PMID: 20304934. doi: 10.1136/bmj.c1066.
30. Hansen TM, Helland Y, Augestad LA, Rand K, Stavem K, Garratt A. Elicitation of Norwegian EQ-5D-5L values for hypothetical and experience-based health states based on the EuroQol Valuation Technology (EQ-VT) protocol. BMJ Open. 2020 Jun 11;10(6):e034683. PMID: 32532768. doi: 10.1136/bmjopen-2019-034683.
31. Gandek B, Ware JE, Aaronson NK, Apolone G, Bjorner JB, Brazier JE, Bullinger M, Kaasa S, Leplege A, Prieto L, Sullivan M. Cross-validation of item selection and scoring for the SF-12 Health Survey in nine countries: results from the IQOLA Project. International Quality of Life Assessment. J Clin Epidemiol. 1998 Nov;51(11):1171-8.
32. Eriksen S, Bjorklof GH, Helvik AS, Larsen M, Engedal K. The validity of the hospital anxiety and depression scale and the geriatric depression scale-5 in home-dwelling old adults in Norway(). J Affect Disord. 2019 Sep 1;256:380-5.
33. Gould R, Ilmarinen J, Järvisalo J. Dimensisons of work ability. Results from the 2000 survey. . Helsinki, Finland.: Finnish Centre for Pensions, 2008, 2008.
34. Smith ORF, Alves DE, Knapstad M, Haug E, Aaro LE. Measuring mental well-being in Norway: validation of the Warwick-Edinburgh Mental Well-being Scale (WEMWBS). BMC Psychiatry. 2017 May 12;17(1):182.
35. Lai JS, Cella D, Choi S, Junghaenel DU, Christodoulou C, Gershon R, Stone A. How item banks and their application can influence measurement practice in rehabilitation medicine: a PROMIS fatigue item bank example. Arch Phys Med Rehabil. 2011 Oct;92(10 Suppl):S20-7. PMID: 21958919. doi: 10.1016/j.apmr.2010.08.033.
36. Amtmann D, Cook KF, Jensen MP, Chen WH, Choi S, Revicki D, Cella D, Rothrock N, Keefe F, Callahan L, Lai JS. Development of a PROMIS item bank to measure pain interference. Pain. 2010 Jul;150(1):173-82.
37. Jensen MP, Karoly P, Braver S. The measurement of clinical pain intensity: a comparison of six methods. Pain. 1986 Oct;27(1):117-26. PMID: 3785962.
38. Johansen T, Lund T, Jensen C, Momsen AH, Eftedal M, Oyeflaten I, Braathen TN, Stapelfeldt CM, Amick B, Labriola M. Cross-cultural adaptation of the Work Role Functioning Questionnaire 2.0 to Norwegian and Danish. Work. 2018;59(4):471-8. PMID: 29733048. doi: 10.3233/WOR-182705.
39. Symonds TL, Burton AK, Tillotson KM, Main CJ. Do attitudes and beliefs influence work loss due to low back trouble? Occup Med (Lond). 1996 Feb;46(1):25-32. PMID: 8672790. doi: 10.1093/occmed/46.1.25.
40. Nottingnes C, Fersum KV, Reme SE, Moe-Nilssen R, Morken T. Job-related self-efficacy in musculoskeletal disorders - a questionnaire. Tidsskr Nor Laegeforen. 2019 Aug 20;139(11). PMID: 31429250. doi: 10.4045/tidsskr.18.0571.
41. NICE. NICE guide to the methods of technology appraisal. National Institute for Health & Clinical Excellence (NICE), 2008.
42. Dolan P. Modeling valuations for EuroQol health states. Medical care. 1997 Nov;35(11):1095-108. PMID: 9366889.
43. Brazier JE, Roberts J. The estimation of a preference-based measure of health from the SF-12. Medical care. 2004 Sep;42(9):851-9. PMID: 15319610. doi: 00005650-200409000-00004 [pii].
44. Steckler A, Linnan L. Process evaluation for public health interventions and research. . San Francisco: : Jossey-Bass; 2002.
45. Ritchie J, Lewis J. Qualitative Research Practice: Sage Publications; 2003.
46. Froud R, Patterson S, Eldridge S, Seale C, Pincus T, Rajendran D, Fossum C, Underwood M. A systematic review and meta-synthesis of the impact of low back pain on people's lives. BMC Musculoskelet Disord. 2014 Feb 21;15:50.
47. Grant M, O'Beirne-Elliman J, Froud R, Underwood M, Seers K. The work of return to work. Challenges of returning to work when you have chronic pain: a meta-ethnography. BMJ Open. 2019 Jun 20;9(6):e025743.
