# Supplementary material for: Living with chronic pain and returning to work: lessons learnt from a process evaluation embedded in the ReISE trial
Source: BMJ Open. 2026 Oct 22;16(10):e125090. doi: 10.1136/bmjopen-2026-125090 (PMC13691893; doi:10.1136/bmjopen-2026-125090)
Supplement: Supplementary file 2 [file bmjopen-2026-125090.reviewer_comments.pdf]

PEER REVIEW HISTORY

BMJ Open publishes all reviews undertaken for accepted manuscripts. Reviewers are asked to complete a checklist review form and are provided with free text boxes to elaborate on their assessment. These free text comments are reproduced below.

ARTICLE DETAILS

Title (Provisional)

Living with chronic pain and returning to work - Lessons learnt from a process evaluation embedded in the ReISE trial

Authors

Irgens, Pernille Marie Stahr; Ellard, David R; Underwood, Martin; Grotle, Margreth; Holmgard, Jakob Thor Einar; Amundsen, Pål Andre; Højen, Magnus; Burton, Kim; Froud, Robert

---

VERSION 1 - REVIEW

**Reviewer**	**1**
**Name**	**Couban, Rachel**
**Affiliation**	**McMaster University, Michael G. DeGroote Institute for Pain Research and Care**
**Date**	**07-Aug-2026**
**COI**	**None**

An interesting study and hopefully will lead to people being supported to return to work

---

**Reviewer**	**2**
**Name**	**Goodwin, Peter**
**Affiliation**	**MMU, Health Professions**
**Date**	**15-Aug-2026**
**COI**	**None**

Thank you for giving me the opportunity of reviewing this study - Living with chronic pain and returning to work - Lessons learnt from a process evaluation embedded in the ReISE trial

Introduction:

• Page 6, line 24: please clarify what you mean by “a designated coordinating role”.

• Page 6, line 29: when you say “and depend on long-term benefits”, do you mean government provided social financial benefits?

• Page 7, line 9: The aim was to assess recruitment, reach, dose, fidelity, and acceptability of the ReISE intervention, which sounds like a pilot / feasibility study. Was one completed before the trial? How does it fit?

Methods:

• Page 13, line 11: Please clarify how qualitative and quantitative findings were integrated at the interpretation stage and assessed.

Results:

• Page 13, line 37: please put the recruitment of 1,622 into context of your sampling frame – those eligible from the total unemployed in Norway.

• Pge 18, line 49: Please describe how "Communication processes were streamlined during the trial to reduce delays"

• Page 20, line 55: The key to this whole paper is the line "Participants frequently described themselves as motivated". There is strong bias here. What about those who were not motivated? What did yo udo to recruit these? Was motivation considered?

• There is no reference to how many this refers to. "Formal fidelity observations of three work familiarisation session sessions were carried out (by PMI). Both adherence (97-100%; mean 99%) and competence (100%) were seen to have been delivered as intended across all the observations (See Table S5, Supplementary File 1, Part 4)"

• What is the evidence of this? "Among those invited to the intervention, 55% declined. This may reflect the difference between research participation and intervention participation, as well as poor timing, health burden, welfare-related concerns, and exhaustion from previous unsuccessful attempts to return to work." If feel this could have been explored further and action plans produced.

Discussion:

• Page 32, line 9: the statement "the intervention is conceptually sound" does not provide context and is misleading considering such a small number of people who declined the intervention. The conclusion is highly overinflated.

• Page 30, line 19: Limited reflection on capacity when availability was not met in such a small cohort – "Scheduling availability was the most common reported cause of delay between acceptance and completed initial assessment."

• The conclusion is totally misleading. The statement "It demonstrates that a structured, case manager-led return-to-work intervention with placement is deliverable in this population" must reflect the small proportion of the population it included and include "how a substantial proportion of those recruited did not genuinely want to participate in the intervention".

• The study reflects on participant heterogeneity, welfare system conditions, labour market factors, and limited integration between components, not on the demographics of those that did not participate. What will you do to target and increase inclusion of those hard to reach people.

• Were incentives included to recruit participants in the main trial and this study?

• There is a clear segmentation profile here. It does not seem to be addressed or accounted for in the study.

• Supp file 1, Part 1: Was the following a shock – if so why? How did it fit with the pilot or feasibility study? “Allocation ratio was initially set at 1:3 but was adjusted to 2:1 during the study period to ensure sufficient intervention throughput despite lower-than-anticipated acceptance rates.”

VERSION 1 - AUTHOR RESPONSE

B. Reviewer: 1

Comments to the Author:

An interesting study and hopefully will lead to people being supported to return to work

Thank you for your encouraging comments. We hope that these findings will contribute to improving return-to-work support for people living with persistent pain.

C. Reviewer: 2

Comments to the Author:

Thank you for giving me the opportunity of reviewing this study - Living with chronic pain and returning to work - Lessons learnt from a process evaluation embedded in the ReISE trial

Introduction:

1. Page 6, line 24: please clarify what you mean by “a designated coordinating role”.

We appreciate the feedback on clarifying this description. We have defined the coordinating role at its first mention in the introduction and explained its intended relation to the ReISE case manager.

2. Page 6, line 29: when you say “and depend on long-term benefits”, do you mean government provided social financial benefits?

Yes, we refer to public income-replacement benefits. We have clarified this in the Introduction and defined the Norwegian Labour and Welfare Administration (NAV) at its first mention. The revised wording does not imply that all unemployed people receive these benefits.

3. Page 7, line 9: The aim was to assess recruitment, reach, dose, fidelity, and acceptability of the ReISE intervention, which sounds like a pilot / feasibility study. Was one completed before the trial? How does it fit?

The trial included an internal pilot phase which was completed. We have expanded the description to explain the basis for progression to the full trial and the uptake and placement difficulties identified during the pilot. The process evaluation presented here examines implementation and participant and staff experiences across the trial, including the proposed processes supporting engagement and progression to placement.

Methods:

4. Page 13, line 11: Please clarify how qualitative and quantitative findings were integrated at the interpretation stage and assessed.

Thank you for this comment. We have updated the text explaining how integration was organised around the process evaluation domains, since the qualitative framework had been coded deductively on those same domains and the two datasets were therefore already structured in the same way. We have expanded that part in the Methods to describe the sequence in which the datasets were analysed, how the quantitative indicators and the charted qualitative accounts were set against one another, how divergence between participant and staff accounts was handled, and that convergence and divergence were judged by discussion between two of the authors rather than by a formal rule. We have also added a worked example. No new analysis was undertaken; the expanded passage describes the procedure that was followed.

Results:

5. Page 13, line 37: please put the recruitment of 1,622 into context of your sampling frame – those eligible from the total unemployed in Norway.

Thank you for pointing out the need to clarify the recruitment context. We have clarified in the Results that the 1,622 people accessed the expression of interest form through an open recruitment process; they were not sampled from an enumerated national population. Available statistics do not identify everyone meeting the ReISE eligibility criteria, so a national population-reach percentage cannot be calculated. We have also added national benefit-recipient comparisons in the Discussion, with the differences in eligibility, age coverage and reference years made explicit. These provide context rather than evidence that the cohort is representative.

6. Page 18, line 49: Please describe how "Communication processes were streamlined during the trial to reduce delays"

Thank you for requesting this clarification. The change involved direct communication between the placement recruiter and case manager, bypassing the trial coordinator. This was already noted among the adaptations, and we have now replaced the general statement in the placement-recruitment paragraph with the specific change. The wording states its intended purpose without claiming that its effect on waiting time was measured.

7. Page 20, line 55: The key to this whole paper is the line “Participants frequently described themselves as motivated”. There is strong bias here. What about those who were not motivated? What did yo udo to recruit these? Was motivation considered?

The original statement referred to interviewees’ accounts of their own motivation. We have revised the wording to make the scope of this finding explicit. Wanting to return to work was an eligibility criterion, and the study therefore cannot represent people who did not want to return to work. However, declining this particular intervention was not an exclusion from the process evaluation: 11 of the 30 interviewees had declined the invitation. The interview guides explored expectations and decisions about participation. We have also clarified that eligibility screening established a general wish to return to work without a specific timeframe, before participants were informed about the possibility of random sampling to be offered the intervention and therefore did not establish willingness to undertake a work placement when invited. We have clarified this inclusion and revised the Results and Discussion to distinguish the wish to work or contribute to research from willingness and capacity to take up the intervention at that time.

8. There is no reference to how many this refers to. “Formal fidelity observations of three work familiarisation session sessions were carried out (by PMI). Both adherence (97-100%; mean 99%) and competence (100%) were seen to have been delivered as intended across all the observations (See Table S5, Supplementary File 1, Part 4)”

Thank you for highlighting that the coverage was unclear. The three observations were reported, but their relation to the total deliveries was not explicit. We have now specified that three of fourteen full sessions were observed and clarified that the adherence and competence percentages describe facilitator delivery in those observations. The Abstract, Results and Table S5 now use the same denominator and terminology.

9. What is the evidence of this? “Among those invited to the intervention, 55% declined. This may reflect the difference between research participation and intervention participation, as well as poor timing, health burden, welfare-related concerns, and exhaustion from previous unsuccessful attempts to return to work.” If feel this could have been explored further and action plans produced.

We see that this could have been made clearer in both the manuscript and Supplementary files. The reasons were explored through invitation contacts recorded in the trial-management log and through interviews, including interviews with people who declined. We have identified these sources in Supplementary File 1 and distinguished the recorded reasons from interview-based interpretation. Because the reasons were volunteered and incompletely recorded, we present them descriptively without estimating their prevalence among all decliners. In the Discussion, the practical recommendations now link these obstacles to clearer information about participation and benefits, flexible timing and opportunities to revisit participation. These are recommendations for future delivery, not actions evaluated during this trial. In addition, we

have revised the text in Supplementary File 1, Part 5, under Mismatch between observational-study participation and intervention readiness, which now reads:

"The cohort-randomised design revealed an important distinction between willingness to participate in an observational study and contribute to research, and readiness to engage in an active work-reintegration intervention. Of the 223 participants invited, 119 (53%) declined the intervention. Reasons volunteered during invitation contacts and recorded in the trial-management log included health difficulties, concerns about benefit implications, timing in relation to other return-to-work measures, and personal circumstances. Interviews with participants who declined provided further accounts of health, timing, practical barriers and previous unsuccessful attempts to return to work. These accounts also indicated that willingness to contribute to research did not necessarily mean willingness or capacity to take up the intervention. The reasons were not collected through a structured questionnaire and were incompletely recorded. Therefore, the records and interviews provide possible explanations for individual decisions, but do not establish the frequency or relative importance of these reasons among all decliners."

Discussion:

10. Page 32, line 9: the statement "the intervention is conceptually sound" does not provide context and is misleading considering such a small number of people who declined the intervention. The conclusion is highly overinflated.

Thank you for this comment. Distinguishing problems of implementation from problems with the intervention itself is a central purpose of a process evaluation, and our intention was to convey that the intervention could be delivered as designed to those who engaged. We accept, however, that the wording overstated what these data can show. We have removed "conceptually sound" and revised the statements attributing effectiveness primarily to implementation conditions. The revised Discussion examines implementation, candidate mechanisms and the assumptions underlying the intervention, without treating these findings as evidence of effectiveness or ruling out limitations in its design. Figure 2 and its legend also now distinguish planned integration with trial outcomes from the work reported here.

11. Page 30, line 19: Limited reflection on capacity when availability was not met in such a small cohort – "Scheduling availability was the most common reported cause of delay between acceptance and completed initial assessment."

Thank you, the point about capacity is helpful. We have expanded the Discussion using the staff accounts already reported in Supplementary File 2. These describe competing duties and uneven demand, which help explain why participant numbers alone do not indicate the time

available for delivery. Participant availability also varied. We have retained the distinction between these possible contributions and what can be established about individual delays.

12. The conclusion is totally misleading. The statement "It demonstrates that a structured, case manager-led return-to-work intervention with placement is deliverable in this population" must reflect the small proportion of the population it included and include "how a substantial proportion of those recruited did not genuinely want to participate in the intervention".

We appreciate the feedback on the conclusion and agree that it might not sufficiently reflect the limited uptake and completion. The revised conclusion now makes it clear that low uptake and reduced progression restricted delivery of the full intervention to a small proportion of invitees, leaving deliverability across the wider eligible population uncertain. The coordinator's interpretation is retained as an attributed staff account in Supplementary File 2 (see below), while the recorded reasons and interview accounts in the Results section now distinguish a wish to return to work from willingness or capacity to undertake this intervention when invited. The Results also clarify that all 22 participants who met the minimum placement adherence threshold completed the full six weeks.

We have also altered the sentence in Supplementary File 2, last box under Participant readiness, motivation, and expectations to: "[TC was the clearest voice on this issue,] interpreting limited uptake as indicating that some cohort participants had not wanted to take part in the intervention itself."

13. The study reflects on participant heterogeneity, welfare system conditions, labour market factors, and limited integration between components, not on the demographics of those that did not participate. What will you do to target and increase inclusion of those hard to reach people.

Thank you for drawing attention to this difficult aspect of our study. Recruitment by open self-referral reaches only those who encounter the study material and act on it, and we think it unlikely that broader advertising alone would have reached the people you have in mind. We have therefore expanded the second point under Practical implications and recommendations in the Discussion to explain why we consider the Norwegian Labour and Welfare Administration to be a key partner for reaching this group, rather than one option among several. The agency administers the benefits this population receives, so recruitment through it could define the eligible population rather than leaving it unknown. It would also reach people irrespective of their use of social media, it would place the offer at the point at which return-to-work measures are already under consideration and could reduce uncertainty about the consequences of taking part for participants' benefits. Two of the reasons given for declining, uncertainty about consequences for benefits and poor timing in relation to other measures, arise within the welfare system and can be addressed only there. We have also noted that such a design would require agreement at agency level and safeguards ensuring that participation remained voluntary.

14. Were incentives included to recruit participants in the main trial and this study?

Thank you for raising this. We have clarified that a voucher was offered for joining the cohort and that no additional payment was offered for accepting the intervention invitation. We have also added a sentence on reimbursement of travel costs. These details have been added to the Methods and Results.

15. There is a clear segmentation profile here. It does not seem to be addressed or accounted for in the study.

We agree that the variation between participants has implications for how support is delivered. This was reflected in the existing discussion of readiness and the recommendation for a stepped or stratified approach. We have now made the basis and limits of that recommendation explicit, drawing on the changes made in response to earlier comments, in particular comment 13: the qualitative accounts indicate differing support needs, but the evaluation did not establish discrete participant profiles or test whether they predicted uptake. Assessment should therefore guide tailoring and timing rather than operate as an additional exclusion criterion. We hope this is now clearly addressed and accounted for.

16. Supp file 1, Part 1: Was the following a shock – if so why? How did it fit with the pilot or feasibility study? "Allocation ratio was initially set at 1:3 but was adjusted to 2:1 during the study period to ensure sufficient intervention throughput despite lower-than-anticipated acceptance rates."

This was not unexpected. Low uptake had already been identified during the internal pilot: eight of the 19 invitees accepted (42%), and allocation to an intervention offer was increased during that phase to maintain throughput. The published protocol anticipated that allocation might need to increase if acceptance was insufficient. We have added this context in Supplementary File 1, Part 1. Study Trial Methodology, while retaining the corrected allocation sequence. We do not imply that every subsequent change was prespecified.

Low uptake had already been identified during the internal pilot, in which eight of 19 invited participants accepted the intervention (42%).1 Allocation to an intervention offer was increased during the pilot to maintain intervention throughput. The published trial protocol also anticipated increasing allocation if acceptance limited throughput.2 Thus, low uptake was an issue identified during piloting rather than first encountered in the full trial.

References

1. Amundsen PA, Underwood M, Burton K, Grotle M, Engedahl MS, Malmberg-Heimonen I, et al. Individual supported work placements (ReISE) for improving sustained return to work in unemployed people with persistent pain: an internal pilot study of a cohort randomised controlled approach. Pilot Feasibility Stud. 2024;10(1):110.

2. Amundsen PA, Underwood M, Burton K, Grotle M, Malmberg-Heimonen I, Kisa A, et al. Individual supported work placements (ReISE) for improving sustained return to work in unemployed people with persistent pain: study protocol for a cohort randomised controlled trial with embedded economic and process evaluations. Trials. 2023;24(1):179.
