# Supplementary material for: Living with chronic pain and returning to work: lessons learnt from a process evaluation embedded in the ReISE trial
Source: BMJ Open. 2026 Oct 22;16(10):e125090. doi: 10.1136/bmjopen-2026-125090 (PMC13691893; doi:10.1136/bmjopen-2026-125090)
Supplement: Supplementary file 3 [file bmjopen-16-10-s001.docx]

**SUPPLEMENTARY FILE 1**

**Table of contents**

Table S1 Details of the researchers involved in the qualitative analysis (Supporting COREQ Checklist) ………………………. 1

Part 1. Study Trial Methodology ……………………………………………………………………………………………………………………………... 2

1.1 Detailed Recruitment Flow and Exclusions …………………………………………………………………………………………2

1.2 Detailed intervention methodology ……………………………………………………………………………………………………2

Part 2. Summary of the Interview Topic Guides for participants and intervention staff ……………………………….............. 4

Table S2 Topic guide participants ……………………………………………………………………………………………………………..4

Table S3 Topic guide intervention staff …………………………………………………………………………………………………………. 5

Part 3. Baseline characteristics of interviewed participants …………………………………………………………………………………….. 6

Table S4 Baseline characteristics of interviewed participants (n=30) ………………………………………………………..6

Part 4. ReISE Work Familiarisation Session Evaluation Form from three observed sessions ……………………………….……. 8

Table S5 Fidelity assessment of three observed Work Familiarisation Sessions ………………………………………..8

Part 5. Detailed Procedural Challenges ………………………………………………………………………………………………………………...... 9

**Table S1 Details of the researchers involved in the qualitative analysis (Supporting COREQ Checklist)**

| **Researcher initials** | **Sex** | **Task performed** | **Occupation** | **Experience** | **Qualification** |
| --- | --- | --- | --- | --- | --- |
| PMI | F | Conducted interviews  Coded and analysed interview data and open-ended survey data | Chiropractor and health academic | Experience and training in  multi-method research;  published qualitative research. | PhD |
| PAA | M | Coded and analysed data | Osteopath and health academic | Experience and training in  multi-method research;  published qualitative research. | PhD |
| DRE | M | Analysed data | Health and process evaluation academic;  Professor emeritus | Extensive experience in multi-method research; published in qualitative research; highly cited researcher | PhD |

**Part 1. Study Trial Methodology**

The ReISE trial employed a pragmatic, single-site, two-stage cohort-randomised controlled design. Initially, participants were recruited into a longitudinal observational cohort to explore the impact of unemployment on individuals with persistent pain. Following baseline measurements, a sub-sample of the cohort was randomly selected and offered a multifaceted return-to-work (RTW) intervention. Allocation ratio was initially set at 1:3 but was adjusted to 1:2 during the pilot, and then further to 2:1 during the study period to ensure sufficient intervention throughput despite lower-than-anticipated acceptance rates.

Low uptake had already been identified during the internal pilot, in which eight of 19 invited participants accepted the intervention (42%).^1^ Allocation to an intervention offer was increased during the pilot to maintain intervention throughput. The published trial protocol also anticipated increasing allocation if acceptance limited throughput.^2^ Thus, low uptake was an issue identified during piloting rather than first encountered in the full trial.

1.1 Detailed Recruitment Flow and Exclusions

Recruitment was conducted over a 24-month period, from June 2022 to June 2024. A high level of initial interest was observed, with 1,622 individuals accessing the digital expression of interest form. Of these, 1,530 completed the form, corresponding to 94%, while a small minority indicated no interest or failed to meet basic inclusion criteria. Inclusion criteria were people aged 18-65 who had been unemployed for at least 1 month, had had pain for more than 3 months, and who wanted to work. Exclusion criteria were insufficient Norwegian or English language skills to give consent. Participants over the age of 65 were excluded as they were considered to have limited future time in the workforce before retirement (aged 67 in Norway).

The transition from interested individual to consented participant represented a significant point of attrition. A total of 1,225 individuals were forwarded to the cohort consent form; however, 406 did not complete this step without giving a cause. Of the 819 who responded to the consent form, 330 were excluded for various reasons: 58 were ineligible after further screening, 38 did not give consent, and 23 were removed as duplicate entries. The largest single loss of potential participants occurred when 199 individuals became uncontactable during this stage.

Ultimately, 489 individuals were eligible and consented to study participation. Following completion of the baseline questionnaire by 458 participants, randomisation was performed. After randomisation, 11 individuals were removed: ten because they were later found to be ineligible and one due to duplicate consent. This left a final study sample of 447 participants: 224 allocated to cohort-only group and 223 to intervention group. Of the 223 randomised to the intervention, we were unable to get in contact with 21 participants, and 119 participants declined the invitation. Reasons volunteered during invitation contacts and recorded in the trial-management log included poor health, concerns about losing benefits through work-placement participation, timing in relation to other return-to-work measures, and personal circumstances. These reasons were not collected through a structured questionnaire and were incompletely recorded; they are therefore presented descriptively without ranking their frequency. This left 83 consenting to participate in the intervention, of which 3 discontinued due to poor health before being assigned a case manager. The patient flow of participants consenting to the intervention is described in Figure 1 in the manuscript.

1.2 Detailed intervention methodology

The ReISE intervention comprised individually tailored case management, a work familiarisation session, and a supported work placement.

***Case manager:*** Following allocation to the intervention, participants were contacted by a trained case manager who conducted an initial assessment by telephone or video following the Psychosocial Flags Framework (see below). The purpose was to explore the participant’s work aspirations, previous experience, perceived skills, and barriers to work participation. Using a biopsychosocial and work-focused approach, the case manager supported the participant in identifying modifiable obstacles to return to work and in considering possible strategies for addressing these.

*The Psychosocial Flags Framework*

The core of the intervention delivery was informed by the Psychosocial Flags Framework. ^3^ This framework is a practical biopsychosocial guide for identifying obstacles to recovery and return to work in people with musculoskeletal and pain-related problems. Rather than focusing solely on diagnosis or symptom history, the framework directs attention to psychosocial factors that may hinder work participation.

The Psychosocial Flags Framework is based on a stepped-care logic. ^4^ This approach is particularly relevant when it is not possible at the outset to determine with confidence which level of care an individual requires. In stepped care, treatment is adjusted over time according to the patient’s response, so that the level of intervention reflects the person’s needs while avoiding unnecessary intensity, complexity, burden, and cost. ^4^ Patients usually begin with the least intensive appropriate option and are moved to a higher level of care only when the initial intervention is insufficient, when the response is poor, or when there is good reason to expect that a low-intensity intervention will not be effective. ^3^ The model also depends on repeated review, allowing care to be increased or reduced as patient’s progress becomes clearer. ^5^

“Flags” are conceptualised as warning signals that factors within the individual, the workplace, or the wider social and welfare context may be acting as barriers to recovery or return to work. These include individual beliefs and expectations, emotional responses, workplace-related barriers, and broader organisational or system-level constraints. ^3^ By identifying these barriers, the framework supported a structured and collaborative dialogue between the case manager and participant, and where relevant the placement provider, to develop a tailored return-to-work plan.

***Work familiarisation session:*** Participants were then invited to attend a two-day work familiarisation session, delivered in small groups by a facilitator and an assistant. The session was designed to prepare participants for work re-entry by addressing work-focused pain management, expectations about working with persistent pain, and practical considerations relevant to entering a workplace after a prolonged period outside employment. Sessions were delivered in person where feasible, and digitally for participants living outside the Oslo area or where digital delivery was more practical.

During the work-familiarisation session, participants were introduced to SMART goal-setting: defining goals that are Specific, Measurable, Achievable, Relevant and Time-bound, ^6^ as a structured approach to formulating and working towards return-to-work and other personal goals, ^7^ and were encouraged to apply this approach independently between sessions. They also completed a "time wheel", mapping how they would ideally allocate a typical day across activity, rest and discretionary time. This exercise was framed prospectively: a plan for how participants wished to use their time rather than a record of current routines, to help them identify capacity for valued activities and structure progress towards their goals.

For participants living in or near Oslo, or where participant and CM lived within commuting distance, physical meetings with CM and at WFS were encouraged and attempted. Where not possible, meetings and sessions were done digitally or over telephone.

***Work placement:*** The intervention also included a six-week, part-time work placement of up to 16 hours per week. The placement was intended to be matched to the participant’s skills, aspirations, health limitations, and practical circumstances. During the original intervention model, placements were to be identified through collaboration with Manpower, a national recruitment company in Norway. However, following the pilot phase, this model was revised because identifying suitable placements through this route proved more difficult than anticipated. A dedicated placement recruiter was therefore employed to work alongside case managers in identifying possible placement opportunities and liaising with potential placement providers.

Once a placement had been identified, the case manager, participant, and placement manager met to agree a workability plan. This plan specified the intended work tasks, working hours, support needs, and any temporary accommodations required to make the placement feasible. Throughout the intervention period, the case manager provided regular support to the participant and, where relevant, to the placement manager, primarily by telephone, video, or text message. The case manager could also facilitate work-focused healthcare input or signpost to other services where this was considered necessary.

**Part 2. Summary of the Interview Topic Guides for participants and intervention staff**

**Table S2 Topic Guide (Participants)**

| **Main points** | **Hints** |
| --- | --- |
| Background and current situation | Work situation before being invited to ReISE; main things affecting possibility of returning to work at that time; how pain, health, energy, daily responsibilities, or NAV/benefits influenced the situation |
| Previous experiences with health services, NAV, and work support | Kinds of support received before ReISE; what had been helpful or unhelpful in previous contact with NAV, healthcare, or employers; whether previous experiences affected expectations or trust in ReISE |
| Invitation to the intervention and decision to accept or decline | Understanding of what ReISE was when invited; what drove the decision to accept, decline, or stop participating; whether the timing was right, or reasons it was difficult to engage then |
| Expectations and readiness for return to work | What the participant hoped the intervention would help with; how ready they felt for work-related activity when invited; whether expectations matched what the intervention actually offered |
| Experience of the case manager | Experience of contact with the case manager; whether they felt understood, listened to, and supported; whether support was practical, emotional, work-focused, or something else |
| Individual tailoring and flexibility | Whether the intervention felt adapted to their situation and needs; times when more flexibility or different support was needed; parts that felt too rigid, too fast, too slow, or not relevant |
| Work familiarisation session | Experience of the work familiarisation session; usefulness of meeting others in a similar situation; whether the session helped reflection on work, barriers, coping, or next steps |
| Work placement process | Experience of finding or being offered a placement; whether the placement matched health, skills, interests, and limitations; concerns that the placement could worsen symptoms, energy, or functioning |
| Coordination and progression through the intervention | How the different parts fit together; delays, unclear information, or transitions affecting motivation; whether they knew who to contact and what would happen next |
| Contextual barriers and facilitators | Outside factors making participation easier or harder; effect of NAV rules, benefit concerns, transport, family responsibilities, health fluctuations, or employer attitudes; what would have needed to be different for the intervention to work better |
| Perceived outcomes and meaning | Whether participating changed anything, even without leading to work; effect on confidence, self-understanding, motivation, social belonging, or views about work; any negative effects, disappointments, or unintended consequences |
| Suggestions for improvement | What should be kept in a future version of ReISE; what should be changed; advice for staff delivering this type of intervention |

**Table S3 Topic Guide (Intervention Staff)**

| **Questions for all intervention staff** | |
| --- | --- |
| **Main points** | Hints |
| Role and experience of delivering ReISE | Role in the intervention; how clear the role was in practice; what worked well, and what was difficult |
| Participant group and readiness | Description of the participants who were invited or took part; whether participants seemed ready for a work-focused intervention; what affected their engagement, motivation, or ability to continue |
| Intervention components and delivery | How the different parts of ReISE worked in practice; components that were especially useful or difficult to deliver; whether adaptations were needed during implementation |
| Coordination and communication | How well communication between staff roles worked; delays or unclear transitions between intervention steps; what would have improved coordination |
| Acceptability and perceived usefulness | How participants seemed to experience the intervention; what seemed most useful for participants; parts that seemed less acceptable, less relevant, or potentially difficult for participants |
| Contextual barriers and facilitators | External factors affecting implementation; how NAV, benefit concerns, employer engagement, or labour-market conditions influenced delivery; which barriers were within or outside the project's control |
| Outcomes and mechanisms | What seemed to help participants move forward; whether changes were mainly related to work, confidence, structure, social contact, self-understanding, or something else; what outcomes should be considered important in this type of intervention |
| Recommendations | What should be kept in a future version of ReISE; what should be changed; what would be needed to implement this intervention more successfully |
| **Specific questions by intervention staff role** | |
| **Case managers** | How did you build trust and support with participants?  How useful were the assessment tools and return-to-work plan?  What made the role difficult, unclear, or too broad?  What support or training would have helped you deliver the role better? |
| **Work familiarisation facilitator** | How did participants respond to the WFS?  What worked best in the session?  Were there parts that caused frustration, distress, or drift away from work-focused discussion?  Could the intervention staff setup be different? If so, how? |
| **Placement recruiter** | What was it like to recruit employers and placements?  What made it easier or harder to find suitable placements?  How well did placements match participants’ needs, health, skills, and expectations?  What would improve the placement component? |
| **Trial coordinator** | What were the main operational challenges in running the intervention?  How did recruitment, consent, scheduling, and participant contact affect implementation?  Where were the main bottlenecks in the intervention pathway?  What changes would be needed for a future trial or implementation? |

**Part 3. Baseline characteristics**

**Table S4 Baseline characteristics of study participants randomised to intervention (n=223)**

| **Baseline characteristic** | **Accepted invitation**  **n=83 (37%)** | **Rejected invitation/unable to contact**  **n=140 (63%)** |
| --- | --- | --- |
| Age, mean (SD) | 45 (10) | 45 (9) |
| Sex, female n (%) | 73 (88) | 120 (86) |
| Duration of pain, n (%) |  |  |
| 3-6 months | 1 (1) | 0 (0) |
| 6-12 months | 1 (1) | 1 (<1) |
| 1-3 years | 7 (8) | 21 (15) |
| 3-10 years | 20 (24) | 46 (33) |
| > 10 years | 54 (65) | 72 (51) |
| Duration not working, n (%) |  |  |
| 1-3 months | 3 (4) | 5 (4) |
| 3-6 months | 3 (4) | 4 (3) |
| 6-12 months | 8 (10) | 9 (6) |
| 1-2 years | 12 (14) | 25 (18) |
| > 2 years | 57 (69) | 97 (69) |
| Perceived duration to return to work, n (%) |  |  |
| < 1 month | 0 (0) | 1 (<1) |
| 1-2 months | 0 (0) | 0 (0) |
| 2-4 months | 3 (4) | 4 (3) |
| 4-10 months | 21 (25) | 20 (14) |
| > 10 months | 59 (71) | 115 (82) |
| Work ability^1^, mean (SD) | 3.1 (2.0) | 2.3 (2.0) |
| Confidence in returning to work^2^, mean (SD) | 4.8 (2.5) | 3.6 (2.5) |

SD, Standard deviation; ^1^Work ability, Work Ability Score is a single item taken from the Work Ability Index with the question: “Assume that your work ability at its best has a value of 10 points. How many points would you give your current work ability?”, Scale 0 = completely unable to work to 10 = Work ability at best ^8,9^; ^2^Confidence RTW is from the question: “Assume that your confidence about working at its best has a value of 10 points. How many points would you give your current level of confidence about working?”, scale 0 not confident at all to 10 = Confidence at its best. No missing data.

**Table S5 Baseline characteristics of interviewed participants (n=30)**

| **Characteristic** | **Value** |
| --- | --- |
| Sex, n (%) | 27 (90) |
| Women | 3 (10) |
| Men |  |
| Age, n (%) | 8 (27) |
| 18-39 years | 22 (73) |
| ≥ 40 years |  |
| Duration of pain, n (%) | 4 (13) |
| 1-3 years | 10 (33) |
| 3-10 years | 16 (53) |
| > 10 years |  |
| Duration not working, n (%) | 10 (33) |
| ≤ 2 years | 20 (67) |
| > 2 years |  |
| Perceived duration to return to work, n (%) | 3 (10) |
| 4-10 months | 27 (90) |
| > 10 months |  |
| Work ability, scale 0-10, median (IQR) | 2.4 (0-6) |
| Confidence in returning to work, | 3.6 (1-10) |
| Intervention participation |  |
| Declined the intervention invitation | 11 (37) |
| Accepted but did not complete the full intervention | 13 (43) |
| Completed the full intervention | 6 (20) |

IQR, Interquartile range, ^1^Work ability, Work Ability Score is a single item taken from the Work Ability Index with the question: “Assume that your work ability at its best has a value of 10 points. How many points would you give your current work ability?”, Scale 0 = completely unable to work to 10 = Work ability at best ^8,9^; ^2^Confidence RTW is from the question: “Assume that your confidence about working at its best has a value of 10 points. How many points would you give your current level of confidence about working?”, scale 0 not confident at all to 10 = Confidence at its best. No missing data.

**Part 4. ReISE Work Familiarisation Session Observation Form from three observed sessions**

**Table S6 Fidelity assessment of three observed work familiarisation sessions, of fourteen delivered. Percentages describe facilitator adherence and competence in the observed deliveries.**

| **Adherence** | | | | | **Competence** | | | | |
| --- | --- | --- | --- | --- | --- | --- | --- | --- | --- |
| **Item** | Adherence | Evaluation | | | Item | Competence measure | Evaluation | | |
| Did the facilitator(s): |  | I | II | III | Did the facilitator(s): |  | I | II | III |
| Introduce the session and introduce participants and facilitators? | Yes (2) Partially (1) No (0) | 2 | 2 | 2 | Create opportunities for and encourage discussion and participation in discussion? | Evident (2) Partially evident (1) Not evident (0) NA | 2 | 2 | 2 |
| Go through goals of the sessions? | Yes (2) Partially (1) No (0) | 2 | 2 | 2 | Encourage a balance of participation between quiet and dominant participants? | Evident (2) Partially evident (1) Not evident (0) NA | 2 | 2 | 2 |
| Present and explain the time-wheel? | Yes (2) Partially (1) No (0) | 2 | 2 | 2 | Encourage individual disclosure? | Evident (2) Partially evident (1) Not evident (0) NA | 2 | 2 | 2 |
| Go through benefits of being part of workforce? | Yes (2) Partially (1) No (0) | 2 | 2 | 2 | Validate participants’ disclosures? | Evident (2) Partially evident (1) Not evident (0) NA | 2 | 2 | 2 |
| Go through self-reflection and it’s purpose? | Yes (2) Partially (1) No (0) | 2 | 2 | 2 | Give encouraging feedback on participants behaviour and/or participation? | Evident (2) Partially evident (1) Not evident (0) NA | 2 | 2 | 2 |
| Present exercise of work-related self-reflection and resources and use break-out rooms? | Yes (2) Partially (1) No (0) | 2 | 2 | 2 | Foster a positive, yet realistic, group climate? | Evident (2) Partially evident (1) Not evident (0) NA | 2 | 2 | 2 |
| Explain usual pathway for return-to-work compared to ReISE intervention? | Yes (2) Partially (1) No (0) | 1 | 2 | 2 | Provide appropriate acknowledgements to disclosures or admissions of low self-efficacy? | Evident (2) Partially evident (1) Not evident (0) NA | 2 | 2 | 2 |
| Show video on the self and persistent pain? | Yes (2) Partially (1) No (0) | 2 | 2 | 2 | Provide appropriate acknowledgements to disclosures of negative events? | Evident (2) Partially evident (1) Not evident (0) NA | 2 | 2 | 2 |
| Go through obstacles to work and use break-out rooms? | Yes (2) Partially (1) No (0) | 2 | 2 | 2 | Practice flexibility of time management depending on participants’ previous experience? | Evident (2) Partially evident (1) Not evident (0) NA | 2 | 2 | 2 |
| Explain pain management and show video? | Yes (2) Partially (1) No (0) | 2 | 2 | 2 |  |  |  |  |  |
| Explain balance between health, life, and work? | Yes (2) Partially (1) No (0) | 2 | 2 | 2 |  |  |  |  |  |
| Redo the time-wheel towards end of second day? | Yes (2) Partially (1) No (0) | 2 | 2 | 2 |  |  |  |  |  |
| Go through stories of Petter and Aisha, and have group discussion? | Yes (2) Partially (1) No (0) | 2 | 2 | 2 |  |  |  |  |  |
| Go through the SMART method? | Yes (2) Partially (1) No (0) | 2 | 2 | 2 |  |  |  |  |  |
| Total adherence score (max 28) | | 27 | 28 | 28 | Total competence score (max 18) | | 18 | 18 | 18 |
| Percentage adherence score (*/28x100) | | 97 | 100 | 100 | Percentage competence score (*/18x100) | | 100 | 100 | 100 |
| Mean score from 3 evaluations | | 99 | | | Mean score from 3 evaluations | | 100 | | |

SMARTØF, The SMART method: S – Specific, M – Measurable, A – Achievable, R – Relevant, T – Time-Bound

**Part 5. Detailed Procedural Challenges**

External recruitment

The trial protocol originally relied on an external recruitment agency to source work placements. However, placements were not generated in-line with the needs of the study. This necessitated a major procedural adaptation: the project recruited and trained a dedicated placement recruiter. This change preserved the viability of the placement component but required redistribution of project resources and the development of new internal placement-sourcing workflows.

Resource demands in the invitation process

The process of inviting randomised cohort members to the intervention was more resource-intensive than anticipated. Reaching participants often required repeated contact attempts. In addition, invitation calls were not simple administrative notifications; they frequently lasted 15 to 30 minutes, as staff had to clarify the intervention, respond to concerns, and address uncertainty about potential implications for welfare benefits. This created substantial workload pressure and increased the time required to move participants from randomisation to intervention consent.

Mismatch between observational-study participation and intervention readiness

The cohort-randomised design revealed an important distinction between willingness to participate in an observational study and contribute to research, and readiness to engage in an active work-reintegration intervention. Of the 223 participants invited, 119 (53%) declined the intervention. Reasons volunteered during invitation contacts and recorded in the trial-management log included health difficulties, concerns about benefit implications, timing in relation to other return-to-work measures, and personal circumstances. Interviews with participants who declined provided further accounts of health, timing, practical barriers and previous unsuccessful attempts to return to work. These accounts also indicated that willingness to contribute to research did not necessarily mean willingness or capacity to take up the intervention. The reasons were not collected through a structured questionnaire and were incompletely recorded. Therefore, the records and interviews provide possible explanations for individual decisions, but do not establish the frequency or relative importance of these reasons among all decliners.

Fragmentation and coordination of intervention components

Ensuring a seamless transition between the initial assessment, the two-day work familiarisation session, and the work placement proved difficult. Logistical delays, often related to the time required to identify a suitable placement, created waiting periods between components. These delays could reduce momentum after the work familiarisation session and, for some participants, coincided with deteriorating health or changing life circumstances. This complicated progression into placement highlighted the importance of timing, coordination, and continuity in the intervention pathway.

1.	Amundsen PA, Underwood M, Burton K, Grotle M, Engedahl MS, Malmberg-Heimonen I, et al. Individual supported work placements (ReISE) for improving sustained return to work in unemployed people with persistent pain: an internal pilot study of a cohort randomised controlled approach. Pilot Feasibility Stud. 2024;10(1):110.

2.	Amundsen PA, Underwood M, Burton K, Grotle M, Malmberg-Heimonen I, Kisa A, et al. Individual supported work placements (ReISE) for improving sustained return to work in unemployed people with persistent pain: study protocol for a cohort randomised controlled trial with embedded economic and process evaluations. Trials. 2023;24(1):179.

3.	Kendall NAS, Burton AK, MAin CJ, Watson PJ. Tackling Musculoskeletal Problems: A Guide for Clinic and Workplace—Identifying Obstacles Using the Psychosocial Flags Framework. London, UK: The Stationary Office; 2009.

4.	Von Korff M, Moore JC. Stepped care for back pain: activating approaches for primary care. Ann Intern Med. 2001;134(9 Pt 2):911-7.

5.	Waddell G, Burton A, Kendall N. Vocational rehabilitation: what works, for whom, and when? London: Department for Work and Pensions; 2008. p. 307 p.

6.	Doran GT. There’s a SMART Way to Write Management’s Goals and Objectives. Journal of Management Review. 1981;70:35-6.

7.	Tan V, Cheatle MD, Mackin S, Moberg PJ, Esterhai JL, Jr. Goal setting as a predictor of return to work in a population of chronic musculoskeletal pain patients. Int J Neurosci. 1997;92(3-4):161-70.

8.	El Fassi M, Bocquet V, Majery N, Lair ML, Couffignal S, Mairiaux P. Work ability assessment in a worker population: comparison and determinants of Work Ability Index and Work Ability score. BMC Public Health. 2013;13:305.

9.	Adel M, Akbar R, Ehsan G. Validity and reliability of work ability index (WAI) questionnaire among Iranian workers; a study in petrochemical and car manufacturing industries. J Occup Health. 2019;61(2):165-74.
