# Supplementary material for: Living with chronic pain and returning to work: lessons learnt from a process evaluation embedded in the ReISE trial
Source: BMJ Open. 2026 Oct 22;16(10):e125090. doi: 10.1136/bmjopen-2026-125090 (PMC13691893; doi:10.1136/bmjopen-2026-125090)
Supplement: Supplementary file 4 [file bmjopen-16-10-s002.docx]

**SUPPLEMENTARY FILE 2**

**Detailed qualitative interview findings**

This supplementary file provides a more detailed account of the qualitative interview findings from the process evaluation conducted alongside the ReISE trial. A summary of these findings is reported in the Results section of the main article, where participant and staff accounts are synthesised under seven organising themes. The material presented below expands on those themes by providing fuller analytic summaries and illustrative quotations from study participants and intervention staff.

The qualitative component comprised semi-structured, one-to-one interviews with study participants invited to the intervention and with intervention staff involved in delivery. Thirty participants were purposively sampled after their 12-month follow-up to capture variation in gender, age, whether they accepted or declined the intervention invitation, and the intervention stage reached (invitation, case manager, work familiarisation session, offer of placement, placement, finished placement). All intervention staff were invited and interviewed, including case managers, the work familiarisation session facilitator, the placement recruiter, and the trial coordinator. Interviews explored experiences of intervention delivery and participation, acceptability, perceived usefulness, barriers and facilitators, and contextual factors affecting implementation.

Interview data were managed in NVivo and analysed using the Framework method, combining deductive coding based on the process evaluation domains with inductive coding of themes emerging from the data. This approach was selected because the process evaluation included predefined domains while also requiring comparison across participant pathways, intervention stages, and staff roles. A subset of transcripts was independently coded by two authors, with coding differences and interpretation of candidate mechanisms discussed until consensus was reached. Data were then charted into framework matrices to support comparison across participants who declined, discontinued, or completed the intervention, and across staff roles. The approach follows established guidance for framework analysis in applied health research.

The findings are presented under the same seven organising points as the qualitative results in the main article: the role of the case manager, individualisation and structural constraints, participant readiness and expectations, intervention structure and coordination, the work familiarisation session, work placement experiences, and outcomes beyond employment. Quotations are used to illustrate the analytic points and to preserve differences in perspective between participants and intervention staff. Staff quotations are labelled according to role: CM, case manager; WFS, work familiarisation session facilitator; PR, placement recruiter; and TC, trial coordinator.

**Organising points from the qualitative results:**

1. Role of the Case Manager: Central but Differently Experienced………………………………………………………………………………………………………………………………………………. 2

2. Need for Individualisation vs Structural Constraints…………………………………………………………………………………………………………………………………………..……………………. 3

3. Participant Readiness, Motivation, and Expectations…………………………………………………………………………………………………………………………………………………………….… 3

4. Intervention Structure and Coordination……………………………………………………………………………………………………………………………………………………………………………...… 5

5. Work Familiarisation Session: Reflection vs Drift………………………………………………………………………………………………………………………………………………….…………….…… 7

6. Work Placement: Divergent Experiences but Shared Challenges……………………………………………………………………………………………………………………………………….… … 8

7. Outcomes: Employment vs Broader Gains………………………………………………………………………………………………………………………………………………………………………….…… 9

**Table S6 Detailed qualitative interview findings**

| **Main point from results** | **Detailed text** | **Quotes** |
| --- | --- | --- |
| **Role of the Case Manager: Central but Differently Experienced** | The relationship with the case manager was described by most participants as the single most important component of the intervention. Where the relationship worked well, it was characterised by trust, openness and a sense of being understood - a feeling of being seen and heard in a way that differed from previous experiences with NAV and the healthcare system. | "I thought she was very good. We had a good communication - very open and direct." (P02)  "He was a really good support. It was really good to have someone to lean on." (P20)  "It was very goal-oriented, in a way you could wish for. And he understood me." (P06) |
|  | CM/WFS: The CM group had varied professional backgrounds, including clinical work with musculoskeletal conditions, research, and occupational health. A shared motivation was genuine interest in helping a difficult population, and most found the role professionally meaningful. At the same time, the role was new and poorly defined from the outset - most described having to define it themselves as the project progressed. There was tension between following the manual and exercising professional judgement; the manual was experienced by several as too rigid and insufficiently adapted to the human dimension of the work. The boundary between the case manager role and a therapeutic role was described as artificial and difficult to maintain. WFS emphasised that a physiotherapy background was directly relevant for meeting participants as whole persons. | "I did not follow this manual slavishly. You cannot have a script that covers everything." (CM1)  "The times when you had participants who were genuinely interested and motivated, that was great. Because then you feel you can actually help them." (CM2)  "PMI: Do you think it is important that you see the person, and feel for this group - that it matters to be a physiotherapist, or to have that background? WFS: One hundred percent. Absolutely." (WFS) |
|  | CM/WFS: Relationship-building was described as a prerequisite for everything else in the intervention. Establishing trust took time and was seen as necessary before a meaningful work plan could be developed. Several CMs described navigating the balance between being a supportive and approachable contact person and maintaining a degree of structure and expectation. WFS noted that the digital format limited opportunities for the kind of informal interaction through which trust is naturally built. | "The goal was to establish some kind of contact and relationship and trust. And to build a kind of alliance. Between me and the participant." (CM1)  "I could be a contact person they felt safe with. Rather than having to be the strict one who put their foot down." (CM5) |
| **Need for Individualisation vs Structural Constraints** | Adaptation to individual needs was highlighted as central. Participants who experienced their case manager as attentive to their specific situation - health-related, practical and motivational - consistently reported better experiences. Where the case manager was perceived as rigid or insufficiently tailored, this was described as a barrier. |  |
|  | CM/WFS: There was broad agreement that training was information-heavy but insufficiently operational. The role felt unclear from the start, and several described spending considerable time simply understanding what was expected of them. The manual was experienced as difficult to apply in practice, and training did not adequately prepare CMs for participants with complex mental health needs. One CM felt the training was not adapted to the Norwegian socio-cultural context. Learning during group supervision sessions held throughout the project was described as more valuable than the initial training. WFS highlighted that having a confirmed work placement prior to the course would have made the work preparation sessions more targeted and relevant. | "There was a lot of information… but it was hard to grasp what my role actually was." (CM1)  "The information about how things would unfold, and how intensive the follow-up would be - that was quite limited." (CM2)  "In the absence of proper training, I think it was quite demanding with trial and error." (CM5) |
| **Participant Readiness, Motivation, and Expectations** | Participants' entry into the intervention was characterised by considerable variation in motivation, expectations and life circumstances. Common to most was a genuine desire to return to work, combined with a history of unsuccessful attempts through NAV and the healthcare system. Many had been through multiple measures without progress and described themselves as exhausted by the system - yet still willing to try something new.  Among those who accepted the intervention, curiosity, the research affiliation, and the fact that participation was voluntary and cost-free were highlighted as triggers. Several described having found the study through internet searches, organisations, or social media. Expectations varied: some hoped for direct help finding employment, others wanted tools and support, and some were primarily motivated by contributing to research or helping others in similar situations. | "I just want to do a small service for those who come after me." (P20)  "I have always had a drive to want to come back." (P18)  "It suited me, and it did not cost me a penny to take part." (P16) |
|  | Interviewees who declined the intervention invitation described timing and health difficulties as common reasons for their decision. Some felt unable to manage participation, or that their life situation did not allow it. Logistical barriers - particularly geography - also played a role. Some declined because they did not see themselves as the right target group, or because previous negative experiences with similar measures had undermined their trust that this would be different. | "But it just became completely wrong for me in Oslo." (P10)  "So not all the questions I feel really hit the mark for me." (P21)  "So that it was probably just a good attempt to persuade after all, even if it helps the study - I did not think that was great, really." (P08) |
|  | Early dropout was in most cases caused by deterioration in health or changes in life circumstances that made continued participation impossible - not dissatisfaction with the intervention as such. | *“Doing more would have harmed me in many ways. So I would get worse or not be able to participate in family life.” (P27)* |
|  | CM/WFS: Participants' motivation, self-awareness, and expectations were described as the single factor most strongly influencing delivery and outcome. Many participants had unrealistic expectations - either of themselves or of what the project would deliver. Some overestimated their own competence and readiness for work, making it difficult for CMs to set realistic goals. Participant heterogeneity was considerable: some were highly motivated and reflective, while others presented with very specific and inflexible demands. The gap between expressed motivation at enrolment and actual engagement during the intervention was a recurring theme. | "They cannot see themselves clearly. And not least, they overestimate their own competence and experience." (CM2)  "Many perhaps had slightly too high expectations of what this project would lead to." (CM2)  "It really depends on what kind of participant you are dealing with." (CM5) |
|  | CM/WFS: The cohort composition was described as challenging. Several CMs noted that many participants had agreed to take part without fully understanding what they were committing to, and that inclusion criteria may not have adequately captured readiness for participation. The range of participant profiles within the cohort was described as very wide, creating difficulties for a single intervention approach. | "It seemed like many had said yes to something they perhaps did not fully understand they were saying yes to." (CM5) |
|  | TC was the clearest voice on this issue, interpreting limited uptake as indicating that some cohort participants had not wanted to take part in the intervention itself. The cohort design was valued for its recruitment advantages, but TC noted that the established cohort was not subsequently utilised - a missed opportunity. TC also reflected that the project would likely have had very different recruitment challenges under a conventional RCT design. | "We have recruited people who do not actually want to participate in the intervention. So we have spent a lot of time managing a cohort and an intervention group who were not genuinely interested in taking part in a research intervention." (TC)  "Established a cohort and… Used… All that time and… And work on… Building a cohort. For it not then to be… Used for something… Over time. A… Missed opportunity." (TC) |
| **Intervention Structure and Coordination** | The intervention was largely delivered digitally - via Zoom, telephone and text message - which was accepted by most participants, though not all. Digital contact was experienced as practical and flexible, particularly for participants with geographical constraints or limited mobility. At the same time, several described the digital format as limiting the depth of relationship-building and missed the opportunity for informal contact and physical presence. | "Interviewer: How did you find doing it digitally? P23: Well, it was actually much easier, in a way." (P23)  "A lot of it I have in a way been through before in relation to NAV and other placements." (P18) |
|  | Frequency and availability of contact varied considerably and was described by several as dependent on participants' own initiative rather than a structured plan. Some missed more proactive follow-up, particularly during periods of low activity or when they felt stuck. Others appreciated being able to make contact when needed without feeling obligated. | "It reminded me a little of NAV - just showing up at an office where nothing happens. I could really have wished he had been closer, that I could have talked to him at an office rather than sitting so far away." (P19)  "I thought it was really good. It was really nice to be able to hear and see others in the same situation." (P20) |
|  | Questionnaires were experienced by some as repetitive, tedious or poorly adapted to their actual situation - particularly when they did not reflect changes over time or capture the complexity of participants' lives. Technical solutions such as BankID were mentioned as a potential barrier, but not an insurmountable one for most. | "It can sometimes be a little difficult to feel that you hit the mark on everything, and that you do not misunderstand the questions." (P15)  "In another way it would have been nice to meet the others. So that was a small thing missing, but otherwise it went fine." (P20) |
|  | The structure and coordination between the intervention's different components - course, case manager and work placement - were described by several as unclear. The transition between the course and the placement took a long time, and communication between case manager, participant and workplace was not always sufficiently coordinated. | "It took a very long time from the course to actually getting out to the workplace." (P04)  "Perhaps a bit more role clarification there. That would have made it even better." (P03) |
|  | Some missed more structure in the follow-up - a clearer plan, more frequent contact and better coordination with the workplace during the placement. Identifying and managing challenges along the way was described by some as insufficient, particularly for participants with complex needs. | "I thought it was really good that I had someone to go to. And there was a bit of, talked about some pain guidance and sort of planning the day and those kinds of things." (P18)  "I would really have preferred to participate at a different time." (P22)  "It was almost a relief to just get it a bit over with, or just get started, really." (P18) |
|  | TC: TC described the role as dominated in practice by ad hoc problem-solving and coordination rather than planned implementation. The job description did not match the actual workload, which largely consisted of covering tasks that other actors failed to deliver. | "The biggest barrier to doing the job - what can I say - that my job description essentially set up, was that we had to run from firefighting to firefighting." (TC) |
|  | CM/WFS: Contact patterns and follow-up intensity varied considerably across CMs, and no shared standard existed - something several identified as a structural problem that should have been resolved at system level. In practice, it was always the CM who initiated contact; participants rarely reached out on their own. Several CMs described participants' unpredictability - linked to illness burden - as a persistent challenge for maintaining continuity. Approaches ranged from structured proactive contact via text and phone, to explicitly handing responsibility back to participants, to contact that naturally tapered off over time. | "It was always driven by me. And then there were some participants who were a bit more active - for better or worse - who would contact me back. But that was the exception." (CM2)  "I was perhaps missing some kind of procedure. How many times. Should you make contact. Because you think, ok." (CM1)  "CM5: It was me. PI: It was you? CM5: Yes. PI: Did anyone contact you regularly during the period? CM5: No. PI: No one who did that? CM5: No." (CM5) |
|  | CM/WFS: Digital delivery via Zoom and telephone was used extensively. Most CMs found this workable, though the relational dimension of physical contact was missed. WFS was initially sceptical about delivering the course digitally but found Zoom functioned adequately in practice. What was most missed was the opportunity to have informal contact, check individual understanding, and build relationships naturally. WFS also noted that being at home may have reduced how deeply participants engaged with the material.  TC: TC did not address digital versus physical delivery as a distinct theme but described logistical challenges in coordinating participants and partners across geographic locations. | "What I missed was the relationship you build. If you have a physical course, you can chat informally and get to know the participants." (WFS)  "The most difficult thing was that I could not check each person's understanding individually, because that takes too much time in that kind of setting." (WFS)  "I think that went really well. Being able to establish contact. The digital world." (CM1) |
|  | TC described the onboarding process as chaotic and largely self-directed. Competence developed through experience rather than formal training, and TC reflected that there are real limits to what can be taught in this type of role. | "You build competence by solving problems. Through working life. It is not something you really get trained in." (TC) |
|  | CM/WFS: Time pressure was a central barrier across the CM group. Most carried the intervention alongside clinical work and teaching, and periods of low activity were followed by sudden peaks with several participants simultaneously. The lack of standardisation in contact and follow-up was experienced as a structural problem that should have been addressed at system level. | "Then suddenly there was a rush where you had to deal with five people at the same time." (CM4)  "There were a lot of those conversations… difficult in an otherwise busy working day." (CM2) |
|  | TC identified the loss of the recruitment company partner - responsible for sourcing work placements - as the single largest organisational barrier. This resulted in TC needing to cover tasks equivalent to an additional 50% position. Implementation never reached a stable operational form, and TC noted that early administrative decisions had disproportionately large consequences for the project's overall shape. | "I think we have had a clear challenge in that we essentially had a partner [the recruitment company] who let us down, or walked away from the contract." (TC)  "That has essentially meant that we have had to try to cover… in any case… a 50% position on top of what we were already doing." (TC) |
|  | PR described the role as under-resourced from the start: too small a position, too many tasks, and insufficient capacity for systematic follow-up with employers. | *“Much more time consuming than described. And we should either have had a list of placements that I could call and arrange. That were ready, sort of. Or we should have been more people. Because I didn’t manage to follow up the employers as good as I believe was needed.” (PR)* |
| **Work Familiarisation Session: Reflection vs Drift** | The course was generally described as meaningful and relevant, particularly for those who had been out of working life for a long time. The group format was valued for the sense of community and recognition of meeting others in similar situations - many described this as a new and positive experience. | "It was very interesting. It was a bit like: Ah, good grief. But it went really well. It was actually quite interesting." (P24)  "I think that was the most important outcome. It was something about making it clear to me - you have someone with you who gives you a little security." (P06) |
|  | However, system frustration among participants characterised the group discussions, and several described the course as at times dominated by complaints about NAV and the healthcare system rather than a solution-focused orientation. This was experienced as a barrier to learning by some, while others felt it was important to have space to express frustration. | "It became system-focused and increased helplessness and frustration." (WFS, corroborated by participant accounts)  "Suddenly you had to talk to a person you had never met before, about being in a similar situation. And who had only bad things to talk about regarding their own experiences with NAV. It was a bit like: Ah, good grief." (P24) |
|  | The content was experienced by some as too general or insufficiently operational - particularly regarding concrete job-search tools and understanding of the Norwegian welfare system. Some missed more practical guidance and a clearer link between the course and the subsequent placement. | "Not everyone knows what to do or how to solve things. It does not come with an instruction manual." (P25)  "There was somewhat limited background information to get me started." (described in relation to course content) (P02) |
|  | CM/WFS: System frustration among participants, directed at NAV and other public institutions, was a pervasive contextual factor that affected engagement throughout the intervention. WFS described the course as dominated by this frustration, which made it difficult to maintain a solution-focused orientation. WFS reflected on whether normalising helplessness was appropriate and felt that the group format amplified negative experiences rather than opening space for solutions. Managing participants' frustration with the system required considerable time and capacity from CMs. | "The course was dominated by system frustration. And I think that is a barrier - or an obstacle - to most people getting something useful out of it." (WFS)  "It became system-focused and increased helplessness or frustration." (WFS) |
| **Work Placement: Divergent Experiences but Shared Challenges** | Among those who specifically declined the work placement after having participated in the course, the refusal was primarily about mismatch between the offered placement and their own interests or capacity - not a lack of motivation for work in general. | "I hope it is the wrong time, but I do not think I will return to working life." (P24)  "They found something more relevant. And it is voluntary work." (P24) |
|  | Experiences of the work placement were the most heterogeneous of all intervention components. Where the placement was a good fit, professionally, practically and socially, the experience was described as positive and meaningful, even without subsequent employment. Social inclusion, experiences of mastery and feeling part of a working environment were highlighted as valuable in themselves. | "The difference between that and not going to work is that you are not, or cannot be, part of a community." (P19)  "I got a lot of freedom in terms of how long I could be there and which days and so on, so that was good." (P18) |
|  | Where the placement did not fit - because it did not match the participant's background, expectations or health situation - the experience was negative and, in some cases, directly damaging to motivation and self-image. Mismatch between participant profile and available placements was a recurring problem. | "He had no plan. Had not managed to plan anything. So already there it was like… Yes." (P04)  "It did not quite match what they thought they were going to get." (CM4, corroborated by participant accounts) |
|  | Employer willingness to accommodate was critical. Where employers were flexible and inclusive, the placement worked well. Where there was a lack of adaptation to health-related needs or no real contact person at the workplace, the placement was experienced as unsuccessful. Coordination between case manager, participant and workplace was not always adequate. | "The communication problem at the start - there were several people to relate to." (P02)  "The people you work with are fantastic and accommodating. And it went fine to be at that workplace." (CM4, corroborated by participant accounts) |
|  | Participants who declined the placement did so primarily because of mismatch between the offered placement and their own wishes or capacity - not a lack of motivation for work in general. |  |
|  | PR described a clearly bounded and practical role: finding work placements for participants, but with limited capacity and resources, carried out alongside studies in a small part-time position. | "It started out as a way to earn a little extra money alongside my studies, a small position where the job was to find work placements for participants in the study." (PR) |
|  | PR received a good initial briefing but found that the theory did not work in practice, particularly in securing genuine commitment from employers regarding placements. | "I got a pretty good walkthrough initially, a good explanation of what was wanted, but in practice it did not work at all." (PR) |
|  | CM/WFS: Matching participants to suitable work placements was described as one of the most challenging aspects of the intervention. Mismatch between participants' expectations, competencies, and available opportunities was common. Some participants were highly inflexible about the type of placement they would accept, refusing alternatives. Where placements worked well, employers were described as accommodating and supportive, but participant non-attendance and early dropout remained a problem even in these cases. | "It did not quite match what they thought they were going to get." (CM4)  "They were fixed on that or nothing." (CM5) |
|  | TC identified the failure of the placement recruitment partner as the most fundamental organisational obstacle in the intervention. The solution to this problem arrived very late in the project period.  PR described the core challenge as finding suitable placements with insufficient time and capacity and noted that securing genuine employer commitment was difficult without more systematic follow-up. In addition, the short duration of the placement (six weeks) was mentioned as a restriction in finding placements. | "Where the challenges came from the participant side - that they were not entirely willing to go along with what had been suggested as a placement." (PR)  “In the other end, there are work places that need a lot of training. And regarding the placement duration, if it had been longer, and a more guarantee from our side perhaps that people do show up. Then it might have been worth for them to invest the time and resources to train a participant.”(PR) |
| **Outcomes: Employment vs Broader Gains** | The overall benefit of the intervention was described across a broad spectrum - from sustained return to work, through increased self-understanding and social belonging, to experiences of stagnation or disappointment. Few described the intervention as without value, but many emphasised that the primary gain was not necessarily directly related to the labour market.  Experiences of mastery, social participation and feeling useful were described by many as the most important gains - regardless of whether they returned to paid employment. The relationship with the case manager was frequently identified as the single factor of greatest positive significance. | "Over time I also see how important it is for me to have a sense of belonging somewhere. To have colleagues and those kinds of things." (P20)  "I have got more answers to things. And it is that I perhaps even more clearly need to listen to my body and take more account of my health." (P02)  "I think it is great. That it is being researched." (P16) |
|  | Shortcomings highlighted included: too little practical guidance on job searching and navigating the welfare system, insufficient coordination between the intervention's components, and too little follow-up during the placement period. The rigidity of the system, particularly NAV, was described by many as a persistent barrier that the intervention did not fully address. | "Not everyone knows what to do or how to solve things. It does not come with an instruction manual." (P25)  "Much better than the NAV system. Because there you stand alone regardless." (P20) |
|  | Among those who did not complete the intervention or declined the placement, the most common reflection was that the timing had not been right, not that the intervention itself was wrong. Several expressed that they would recommend the project to others in a similar situation, or that they would have participated at a different point in time. | "I would rather have a little less and be able to accommodate myself. And get the accommodation I need. Rather than not working at all." (P20)  "They found something more relevant. And it is voluntary work. So there was very little pressure." (P24)  "I do not think I will actually return to working life." (P24) |
|  | CM/WFS: Mastery experiences, large and small, were described as central positive mechanisms. Participants who completed the full course and secured employment were held up as the clearest successes, but gains in social participation, daily structure, and self-confidence were also valued. WFS framed this explicitly: experiencing mastery, control, and new possibilities - even without formal employment - was described as a meaningful outcome in itself. | "If they can in some way experience… I have gained something, or I have a sense of meaning in my life, or I feel I am contributing something useful. Then for my part, that is a success." (WFS)  "They start exercising again perhaps. They become more social. They do other things that had perhaps dropped away." (CM4) |
|  | CM/WFS: The outcome picture was described as highly heterogeneous. Clear successes: participants who completed the full intervention and secured employment, were present but not typical. Social reengagement, renewed daily structure, and restored motivation were described as meaningful intermediate outcomes even where employment was not achieved. Some participants had very high and specific demands that went unmet, contributing to dissatisfaction or early dropout. WFS defined success broadly, centred on mastery and meaning rather than employment status alone. | "She had completed everything. And got a job matched to her level of education and competence. So that is a great success, isn't it." (CM2)  "If they can in some way experience… I have gained something, or I have a sense of meaning in my life, or I feel I am contributing something useful. Then for my part, that is a success." (WFS) |
|  | TC assessed outcomes as strongly shaped by organisational conditions rather than the intervention content alone and reflected on what might have been possible with a better-adjusted pilot period. | "I think we could have done things differently and better there." (TC) |
|  | CM/WFS: CMs were consistent in describing effect as conditional on participants' starting point - motivation, realistic self-understanding, and actual proximity to the labour market. Those closest to the labour market with realistic expectations gained most. The quality of the CM-participant relationship, the suitability of the placement match, and sufficient time for follow-up were identified as the key enabling conditions. WFS emphasised that mastery experiences during the placement period were a key mechanism for further progression. | "It really depends on what kind of participant you are dealing with. Those with a motivated starting point… well… they had a different experience." (CM5)  "Seeing that they actually managed something, that they got something to work - that was an important part." (CM3) |
|  | TC identified conditions for effect as largely structural: sufficient capacity for placement recruitment, stable organisational functioning, and genuine participant motivation at the point of enrolment. Without these foundational conditions, intervention content alone was insufficient.  PR highlighted employer willingness to accommodate and follow through as a critical condition - and one that was difficult to secure without more systematic follow-up capacity than the role allowed. |  |

P, participant; CM, case manager; WFS, work familiarisation session facilitator; PR, placement recruiter; TC, trial coordinator
